# Aldehyde dehydrogenase 2 protects against acute kidney injury by regulating autophagy via the Beclin-1 pathway

**DOI:** 10.1172/jci.insight.138183

**Published:** 2021-08-09

**Authors:** Tonghui Xu, Jialin Guo, Maozeng Wei, Jiali Wang, Kehui Yang, Chang Pan, Jiaojiao Pang, Li Xue, Qiuhuan Yuan, Mengyang Xue, Jian Zhang, Wentao Sang, Tangxing Jiang, Yuguo Chen, Feng Xu

**Affiliations:** Department of Emergency Medicine, Chest Pain Center, Clinical Research Center for Emergency and Critical Care Medicine of Shandong Province, Institute of Emergency and Critical Care Medicine of Shandong University, Key Laboratory of Emergency and Critical Care Medicine of Shandong Province, Key Laboratory of Cardiopulmonary-Cerebral Resuscitation Research of Shandong Province, Shandong Provincial Engineering Laboratory for Emergency and Critical Care Medicine, and Key Laboratory of Cardiovascular Remodeling and Function Research, Chinese Ministry of Education, Chinese Ministry of Health and Chinese Academy of Medical Sciences, The State and Shandong Province Joint Key Laboratory of Translational Cardiovascular Medicine, Qilu Hospital of Shandong University, Jinan, China.

**Keywords:** Nephrology, Autophagy, Hypoxia

## Abstract

The mitochondrial enzyme aldehyde dehydrogenase 2 (ALDH2) catalyzes the detoxification of acetaldehyde and endogenous lipid aldehydes. Approximately 40% of East Asians, accounting for 8% of the human population, carry the E504K mutation in *ALDH2* that leads to accumulation of toxic reactive aldehydes and increases the risk for cardiovascular disease, cancer, and Alzheimer disease, among others. However, the role of ALDH2 in acute kidney injury (AKI) remains poorly defined and is therefore the subject of the present study using various cellular and organismal sources. In murine models, in which AKI was induced by either the contrast agent iohexol or renal ischemia/reperfusion, KO, activation/overexpression of ALDH2 were associated with increased and decreased renal injury, respectively. In murine renal tubular epithelial cells (RTECs), ALDH2 upregulated Beclin-1 expression, promoted autophagy activation, and eliminated ROS. In vivo and in vitro, both 3-MA and Beclin-1 siRNAs inhibited autophagy and abolished ALDH2-mediated renoprotection. In mice with iohexol-induced AKI, ALDH2 knockdown in RTECs using AAV-shRNA impaired autophagy activation and aggravated renal injury. In human renal proximal tubular epithelial HK-2 cells exposed to iohexol, ALDH2 activation potentiated autophagy and attenuated apoptosis. In mice with AKI induced by renal ischemia/reperfusion, ALDH2 overexpression or pretreatment regulated autophagy mitigating apoptosis of RTECs and renal injury. In summary, our data collectively substantiate a critical role of ALDH2 in AKI via autophagy activation involving the Beclin-1 pathway.

## Graphical abstract

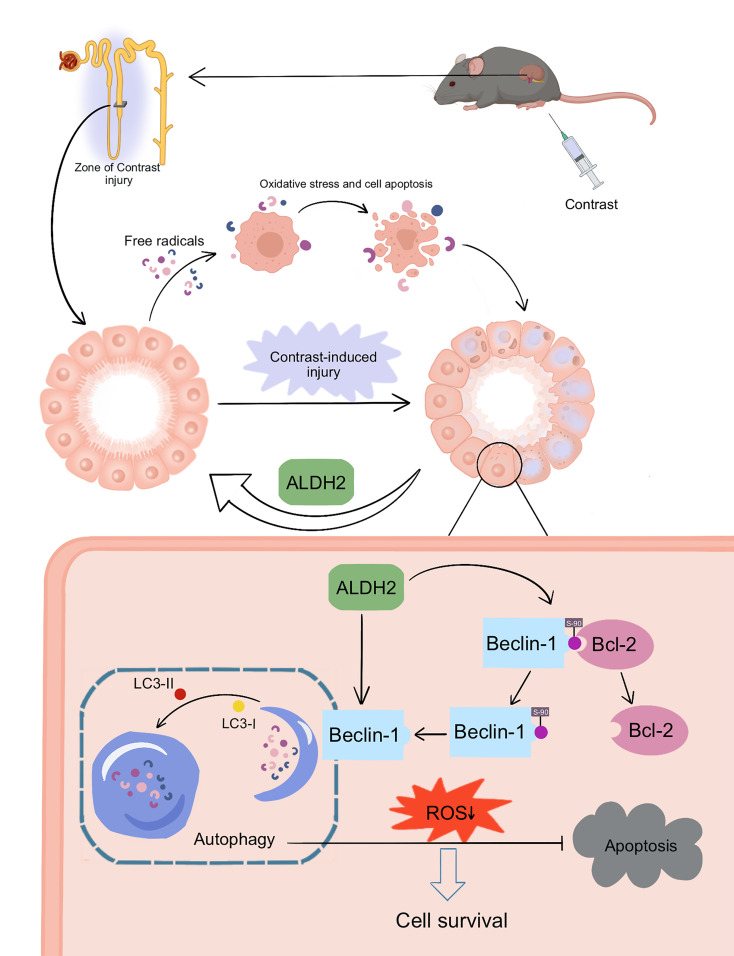

## Introduction

Acute kidney injury (AKI) has been recognized as a major public health problem affecting millions of patients worldwide and leading to decreased survival (1). AKI is usually characterized by a rapid decrease in glomerular filtration rate, with an increase in serum concentration of urea nitrogen, creatinine, and proteinuria (2). AKI is not a single disease but a loose collection of syndromes. Generally, AKI occurs in patients with renal hypoperfusion, cardiovascular disease, sepsis, major surgery, radiocontrast exposure, and pharmacological treatments (3–8). As an important complication, the incidence of AKI in hospitalized patients is approximately 10%–15% and in patients in the intensive care unit its prevalence can sometimes exceed 50% (9–11). Development of AKI in the hospital is associated with an extended length of stay, accelerated onset of end-stage renal disease, increased costs, and increased mortality (12). Moreover, kidney dysfunction or damage can occur over a longer period and accelerate progression of underlying chronic kidney disease (CKD) (10, 13–15). Despite recent insights into the causes and underlying mechanisms, no interventions beyond supportive treatment have been developed to improve outcomes of established AKI (16, 17).

Contrast-induced AKI (CI-AKI) occurs in up to 30% of patients who received iodinated contrast media injection and ranks third among the causes of hospital-acquired AKI, being surpassed only by AKI resulting from decreased renal perfusion (for example, caused by volume contraction, hypotension, or congestive heart failure) and medication-induced impaired renal function (18, 19). Although intravenous saline, sodium bicarbonate, and oral acetylcysteine are frequently used to prevent AKI and adverse outcomes after angiography, said benefit was not seen in recent large-scale randomized control trials, underscoring the need to gain further insight into the pathogenesis of CI-AKI and to develop new treatment and prevention protocols (20–22). In recent studies, CI-AKI and renal ischemia/reperfusion injury (IRI) were documented as major causes of AKI in patients undergoing kidney, liver, or cardiovascular surgery (23). The pathophysiology of CI-AKI and IRI primarily entails distinct but interacting mechanisms, namely renal medullary ischemia and ROS formation (24–27). Within the kidney, hypoxia and the formation of ROS, which play a critical role in AKI pathophysiology via ischemia/reperfusion injury, affect mitochondrial and nuclear DNA, membrane lipids and cellular proteins, and viability and function of renal tubular epithelial cells (RTECs) (28, 29). Furthermore, most risk factors predisposing to AKI tend to enhance renal parenchymal hypoxia and ROS formation. Because scavenging ROS attenuates AKI, reduction of oxidative stress remains an important goal in the prevention of AKI (29, 30).

Aldehyde dehydrogenase 2 (ALDH2) is an allosteric tetrameric enzyme located in the mitochondrial matrix that is abundantly expressed in various organs, such as heart, brain, liver, intestine, and kidney (31–33). ALDH2 is the main enzyme involved in alcohol metabolism and oxidizes various exogenous and endogenous aldehydes into corresponding acids (34–36). A mutant form of *ALDH2*, Lys504, is present in 30%–50% of the East Asian population (present in almost 8% of the global population; refs. 32, 33). The enzyme activity of mutant ALDH2 homozygous and heterozygous genotypes is 17%–38%, respectively, that of the normal ALDH2 genotypes, and presence of the *ALDH2* gene mutation might play a critical role in coronary heart disease, myocardial infarction, heart failure, cancer, and Alzheimer disease, among others (36–42). ALDH2 regulates mitochondrial oxidative ATP production and decreases ROS generation, attenuates the cellular response to oxidative stress both in vitro and in vivo, and protects against myocardial, lung and intestinal ischemia/reperfusion injury, and stroke (43–45). However, the effects of ALDH2 on AKI, especially CI-AKI, pathogenesis remain unclear. Recently, a study identified that inhibition of ALDH2 expression aggravated sepsis-induced renal injury (46). However, another study revealed that continuous use of Alda-1 (an aldehyde dehydrogenase-2 agonist) causes deterioration in renal function following ischemia–reperfusion injury, which is opposite to what had been previously reported for the effects of Alda-1 in cardiac or lung ischemia reperfusion (43, 45, 47). This may be related with impaired renal excretion in AKI rats prolonging Alda-1 exposure leading to increased risk of underlying drug-induced nephrotoxicity, rather than with inhibition of ALDH2. Further, RNA-Seq in previous studies showed markedly lower *ALDH2* mRNA level in kidneys from patients with CKD compared with those from individuals with normal kidney function (controls; refs. 48, 49). However, more evidence on ALDH2 effects in acute and chronic kidney injury should be provided in future.

In this study, we identified a potentially novel mechanism whereby ALDH2 limits ROS levels by promoting autophagy via Beclin-1 upregulation and release from Bcl-2 sequestration contributing to protection of RTECs from oxidative stress and maintenance of kidney hemostasis during CI-AKI and IRI. Therefore, our findings point to a critical role for the ALDH2-Beclin-1 axis in protecting cells from AKI.

## Results

### ALDH2 deficiency aggravated renal injury in CI-AKI mice.

**Figure 1:**
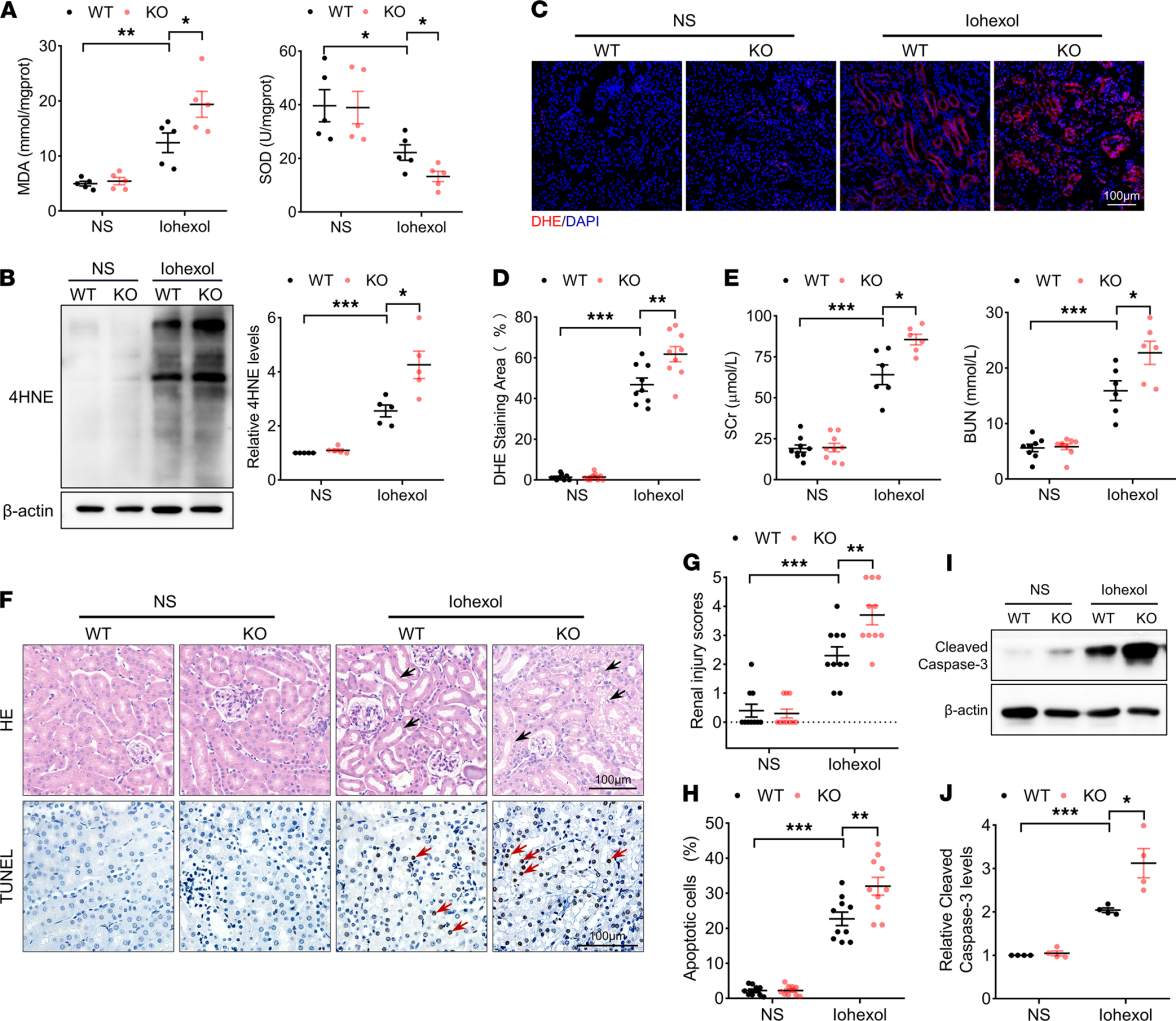
ALDH2 deficiency aggravated renal injury in contrast-induced acute kidney injury mice. (**A**) Malondialdehyde (MDA) and SOD expression in the renal cortex. (**B**) Immunoblotting analysis and quantification of 4HNE in the renal cortex. (**C** and **D**) Representative images and quantification of DHE staining (red) in the renal cortex. Scale bar: 100 μm. (**E**) Renal function was evaluated by SCr (serum creatinine) and BUN (blood urea nitrogen). (**F**–**H**) Representative images and quantification of H&E staining and TUNEL staining in the renal cortex. TUNEL-positive cells are indicated by red arrows. Scale bar: 100 μm. (**I** and **J**) Immunoblotting analysis and quantification of cleaved caspase-3 in the renal cortex. Data are shown as mean ± SEM. Statistical analyses were performed using 1-way ANOVA with a post hoc test (**A**, **B**, **D**, **E**, **H**, and **J**) or χ^2^ test (**G**). *n =* 5–10. *P < 0.05, **P < 0.01, ***P < 0.001.****

To evaluate the role of ALDH2 in CI-AKI, we first established a CI-AKI mice model with iohexol. *ALDH2-*KO mice were constructed to study the role of ALDH2 in CI-AKI (Supplemental Figure 1, A–C; supplemental material available online with this article; https://doi.org/10.1172/jci.insight.138183DS1). After iohexol injection, the level of malondialdehyde (MDA) in the renal cortex significantly increased, whereas that of SOD significantly decreased. In addition, the renal cortex from *ALDH2-*KO CI-AKI mice had a higher level of MDA and a lower level of SOD than that of WT CI-AKI mice (Figure 1A). Immunoblotting analysis revealed that iohexol injection increased 4HNE accumulation in the renal cortex, and to a higher level in *ALDH2-*KO mice (Figure 1B). Similarly, immunofluorescence analysis showed that iohexol-induced ROS accumulation occurred to a higher level in renal tubules of *ALDH2-*KO mice (Figure 1, C and D). In renal function analysis, the levels of serum creatinine (SCr) and blood urea nitrogen (BUN) significantly increased after iohexol injection, and to a greater extent in *ALDH2-*KO mice (Figure 1E). Furthermore, H&E staining was used to better assess injury area and degree of damage of renal tubules subjected to iohexol. Tubular dilation, vacuolar degeneration of tubular epithelial cells, and loss of tubular brush border were apparent in the renal cortex after iohexol injection. Moreover, *ALDH2-*KO mice developed more serious renal morphological injury and higher H&E injury score (Figure 1, F and G). TUNEL staining assay revealed a greater number of apoptotic RTECs in *ALDH2* KO mice than in WT mice after iohexol injection (Figure 1, F and H). Consistent with the results of TUNEL staining, immunoblotting analysis revealed that iohexol injection significantly increased cleaved caspase-3 expression to a greater extent in the renal cortex of WT than *ALDH2-*KO mice (Figure 1, I and J). Therefore, in vivo, *ALDH2* KO increased ROS accumulation and RTECs apoptosis in CI-AKI mice.

### ALDH2 activation attenuated renal injury in CI-AKI mice.

**Figure 2:**
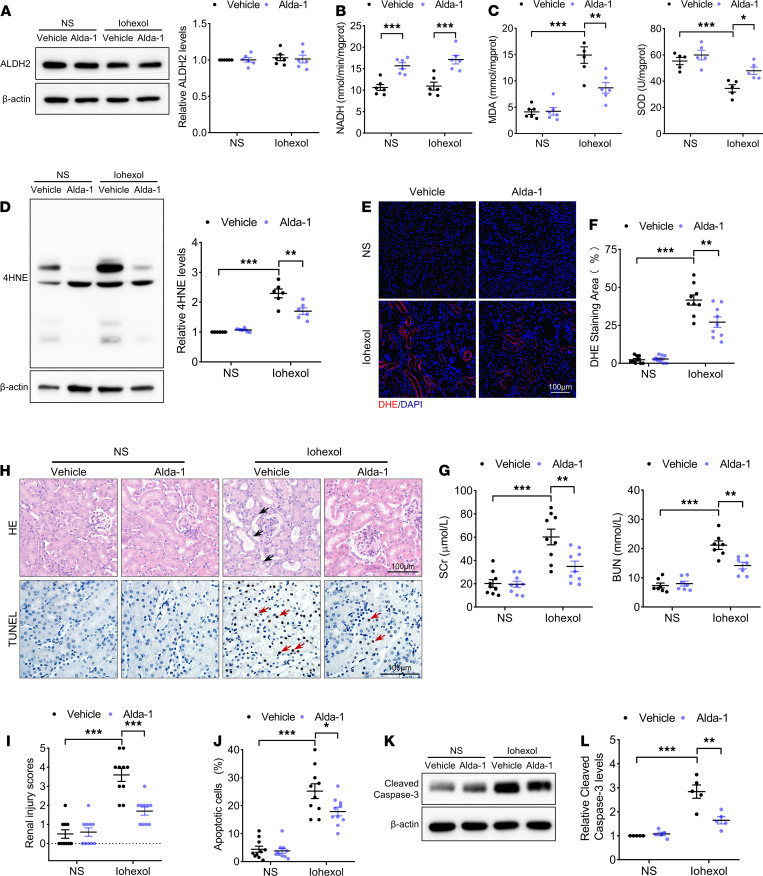
ALDH2 activation attenuated renal injury in CI-AKI mice. (**A**) Immunoblotting analysis and quantification of ALDH2 in the renal cortex. (**B**) ALDH2 enzymatic activity in the renal cortex. (**C**) MDA and SOD expression in the renal cortex. (**D**) Immunoblotting analysis and quantification of 4HNE in the renal cortex. (**E** and **F**) Representative images and quantification of DHE staining (red) in the renal cortex. Scale bar: 100 μm. (**G**) Renal function was evaluated by SCr and BUN. (**H**–**J**) Representative images and quantification of H&E staining and TUNEL staining in the renal cortex. TUNEL-positive cells are indicated by red arrows. Scale bar: 100 μm. (**K** and **L**) Immunoblotting analysis and quantification of cleaved caspase-3 in the renal cortex. Data are shown as mean ± SEM. Statistical analyses were performed using 1-way ANOVA with a post hoc test (**A**–**D**, **F**, **G**, **J**, and **L**) or χ^2^test (**I**). *n =* 5–10. *P < 0.05, **P < 0.01, ***P < 0.001.

Alda-1 was used to study the potential of ALDH2 as a target to prevent against CI-AKI. Injection of only iohexol did not affect the expression and activity of ALDH2 in the renal cortex. However, the renal cortex of WT mice pretreated with Alda-1 showed markedly increased ALDH2 activity (Figure 2, A and B). Alda-1 pretreatment also significantly decreased the MDA level and increased the SOD level in the renal cortex of WT CI-AKI mice (Figure 2C). Moreover, ALDH2 activation attenuated oxidative stress, reduced ROS generation and 4HNE accumulation in the renal cortex of WT CI-AKI mice (Figure 2, D–F). Alda-1 also decreased the levels of SCr and BUN and protected mice from the effects of iohexol, maintaining stable renal function in WT CI-AKI mice (Figure 2G). As shown in H&E staining and renal injury score analysis, Alda-1 pretreatment significantly reduced iohexol-induced renal morphological damage, as reflected by lower degree of tubular dilation and vacuolar degeneration of RETCs (Figure 2, H and I). TUNEL staining revealed that Alda-1 pretreatment significantly decreased the percentage of apoptotic RTECs in WT CI-AKI mice (Figure 2, H and J). Immunoblotting analysis showed that cleaved caspase-3 expression was significantly lower after Alda-1 pretreatment (Figure 2, K and L). Therefore, Alda-1–mediated activation of the ALDH2 enzyme protected RTECs by decreasing ROS production and their apoptosis after iohexol injection.

### ALDH2 activation/OE mediated autophagy activation in RTECs in CI-AKI mice.

**Figure 3:**
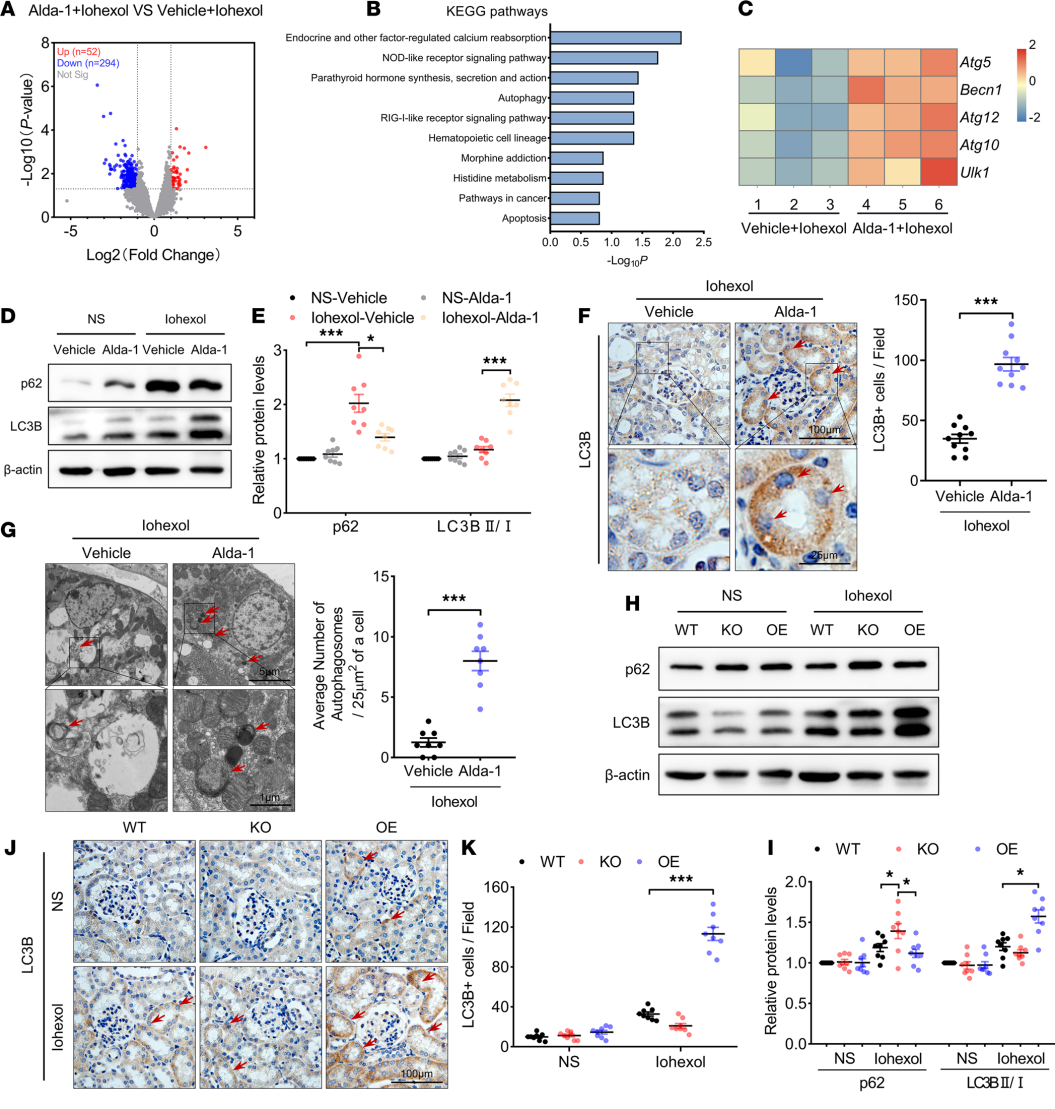
ALDH2 activation/overexpression mediated autophagy activation in renal tubular epithelial cells in CI-AKI mice. (**A**) Volcano plot showing differentially expressed genes due to Alda-1 pretreatment in the renal cortex of CI-AKI mice. The negative log10-transformed *P* values are plotted against the average log2 fold changes in gene expression. Data for genes that are not classified as differentially expressed are plotted in gray. Data for genes that are differentially expressed due to Alda-1 pretreatment (*P* < 0.05) with an absolute log2 fold change of less than or equal to –1 are denoted by blue symbols and data with an absolute log2 fold change of greater than or equal to 1 are denoted by red symbols. (**B**) KEGG pathways enrichment analysis for genes that were classified as differentially expressed due to Alda-1 pretreatment in the renal cortex of CI-AKI mice. A list of the top 10 dysregulated pathways represented by the Alda-1 pretreatment signature. (**C**) Heatmap analysis of differentially expressed transcripts related to autophagy. (**D** and **E**) Immunoblotting analysis and quantification of ALDH2 in the renal cortex. (**F**) Representative images and quantification of immunohistochemical staining of LC3B in the renal cortex. LC3B-positive cells are indicated by red arrows. Scale bar: 100 μm (top), 25 μm (bottom). (**G**) Representative TEM images of autophagosomes and autolysosomes (red arrows) in renal tubular epithelial cells (RTECs). Data are shown as a dot plot of the number of autophagosomes from 10 images of each group. Scale bar: 5 μm (top), 1 μm (bottom). (**H** and **I**) Immunoblotting analysis and quantification of LC3B and p62 in the renal cortex. (**J** and **K**) Representative images and quantification of immunohistochemical staining of LC3B in the renal cortex. LC3B-positive cells are indicated by red arrows. Scale bar: 100 μm. Data are shown as the mean ± SEM. Statistical analyses were performed using 1-way ANOVA with a post hoc test (**E**, **I**, and **K**) or 2-tailed unpaired Student’s *t* test (**F** and **G**). *n =* 8–10. *P < 0.05, **P < 0.01, ***P < 0.001.

To identify pathways potentially mediated by ALDH2, we examined genome-wide transcript level changes in the renal cortex from WT CI-AKI mice. In RNA-Seq analysis, differential gene expression analysis identified increased (52 genes) or decreased (294 genes) mRNA expression with a corrected *P* value of 0.05 and more than a 2-fold change in the renal cortex from Alda-1 pretreatment WT CI-AKI mice (Figure 3A and Supplemental Table 1). Functional enrichment analysis based on KEGG pathway was performed to further elucidate the underlying function of differentially expressed genes (DEGs). The top 10 KEGG pathways are shown in Figure 3B, with a notable number of DEGs enriched in autophagy. Heatmap (Figure 3C) analysis of the transcript expression related to autophagy in the renal cortex indicated that *Becn1/Beclin-1* plays a key role in ALDH2 activation-regulated autophagy. Western blot analysis also indicated that LC3B level was significantly higher in the renal cortex from Alda-1 pretreated WT CI-AKI mice, whereas the p62 level was lower (Figure 3, D and E). Immunohistochemical staining and transmission electron microscopy (TEM) also showed that LC3B level, autophagic vacuoles, and autolysosomes were significantly higher in RTECs from Alda-1 pretreated WT CI-AKI mice (Figure 3, F and G).

*ALDH2*-overexpression (*ALDH2*-OE) mice were used to reconfirm whether ALDH2 mediated autophagy activation in RTECs. The protein levels of ALDH2 were markedly higher in the renal cortex from *ALDH2*-OE mice (Supplemental Figure 1A). Twenty-four hours after iohexol injection, pathological damage of renal tubules, ROS accumulation, and renal function were significantly better in *ALDH2*-OE mice compared with those of WT mice (Supplemental Figure 2). We also evaluated the outcome of iohexol-induced renal injury in *ALDH2-*KO and -OE mice at 48 hours (Supplemental Figure 3). Similar to the 24-hour results, ALDH2 OE protected against renal injury and decreased apoptotic cells, whereas ALDH2 deficiency worsened renal injury. ALDH2 had been shown to prevent the early damage and apoptosis of renal tubular cells and protect against renal injury in AKI. Additionally, LC3B protein levels were remarkably higher in RTECs in *ALDH2*-OE CI-AKI mice, whereas p62 accumulation was significantly lower (Figure 3, H–K). These findings suggest a direct relationship between elucidated that the renoprotection by ALDH2 and autophagy activation.

### Inhibiting autophagy blocked the renal protection of ALDH2 in CI-AKI mice.

**Figure 4:**
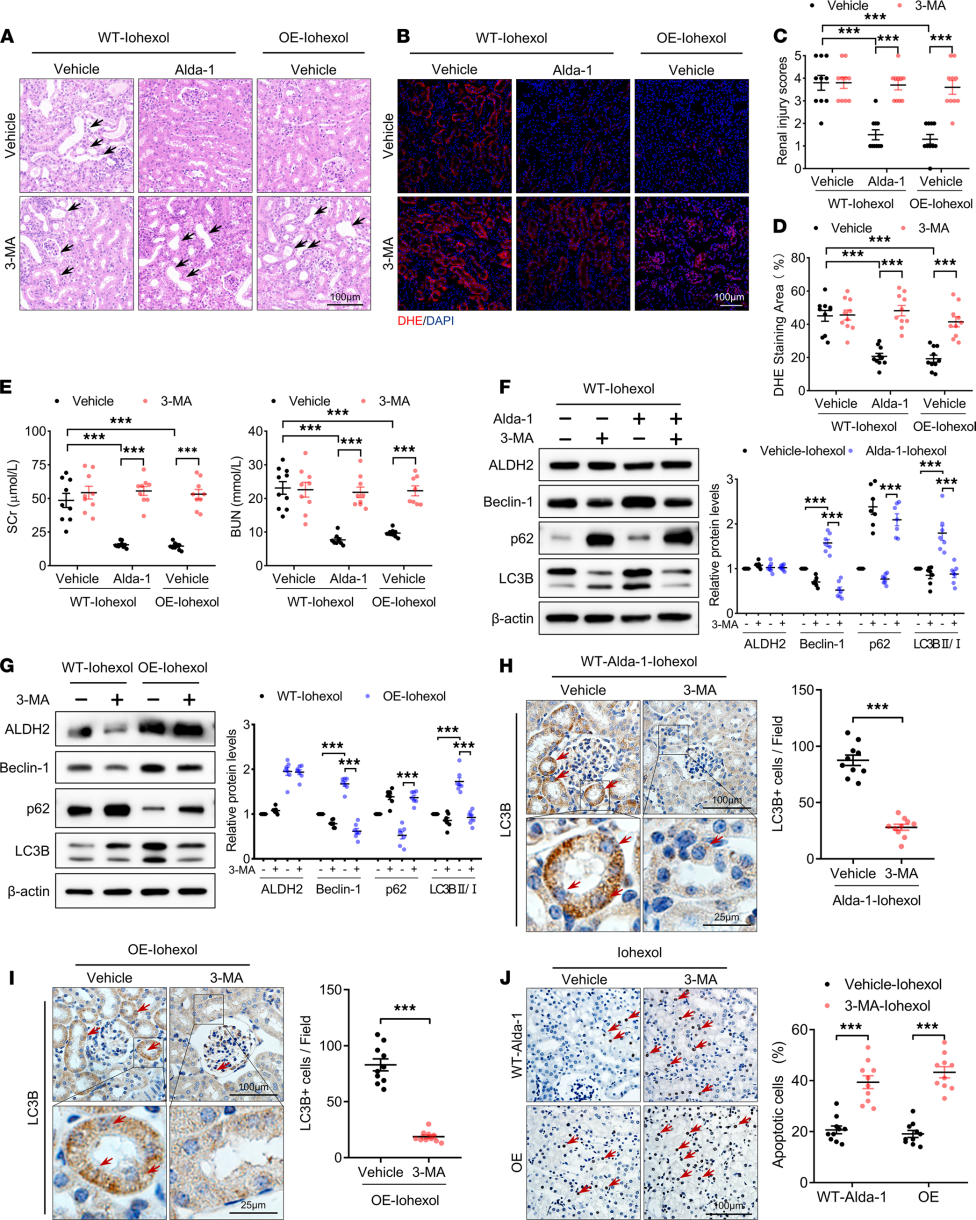
Inhibiting autophagy blocked renoprotection of ALDH2 in CI-AKI mice. (**A**–**D**) Representative images and quantification of H&E staining and DHE staining in the renal cortex. Scale bar: 100 μm. (**E**) Renal function was evaluated by SCr and BUN. (**F** and **G**) Immunoblotting analysis and quantification of ALDH2, Beclin-1, p62, and LC3B in the renal cortex. (**H** and **I**) Representative images and quantification of immunohistochemical staining of LC3B in renal cortex. LC3B-positive cells are indicated by red arrows. Scale bar: 100 μm (top), 25 μm (bottom). (**J**) Representative images and quantification of TUNEL staining in the renal cortex. TUNEL-positive cells are indicated by red arrows. Scale bar: 100 μm. Data are shown as the mean ± SEM. Statistical analyses were performed using the χ^2^ test (**C**), 1-way ANOVA with a post hoc test (**D**–**G** and **J**), or 2-tailed unpaired Student’s *t* test (**H** and **I**). *n =* 7–10. *P < 0.05, **P < 0.01, ***P < 0.001.

As previously described, ALDH2 activation or OE-mediated autophagy activation, decreased ROS production, maintained stable renal function, and reduced apoptosis of RTECs after iohexol injection. The involvement of autophagy in the effect of ALDH2 on CI-AKI was further examined using the autophagy inhibitor 3-methyladenine (3-MA). Compared with vehicle pretreatment, morphological tubular damage and ROS accumulation were higher in 3-MA pretreated ALDH2 activation or OE CI-AKI mice, whereas the increase was not statistically significant in WT CI-AKI mice (Figure 4, A–D). Similarly, 3-MA also significantly increased SCr and BUN levels in ALDH2 activation or OE CI-AKI mice. However, there was no significant difference between WT CI-AKI mice treated or not with 3-MA (Figure 4E). Additionally, autophagic activity was assessed using immunoblotting, and ALDH2 activation or OE increased Beclin-1 and LC3B levels and decreased p62 levels, whereas inhibition of autophagy by 3-MA significantly blocked the latter changes (Figure 4, F and G). Immunohistochemical staining of LC3B also confirmed that 3-MA significantly decreased LC3B in RTECs of ALDH2 activation or OE mice (Figure 4, H and I). Moreover, the inhibition of autophagy by 3-MA largely abolished the protective effect of ALDH2 on RTECs and increased the percentage of apoptotic RTECs in ALDH2 activation or OE CI-AKI mice (Figure 4J).

### ALDH2 OE promoted autophagy activation in RETCs.

**Figure 5:**
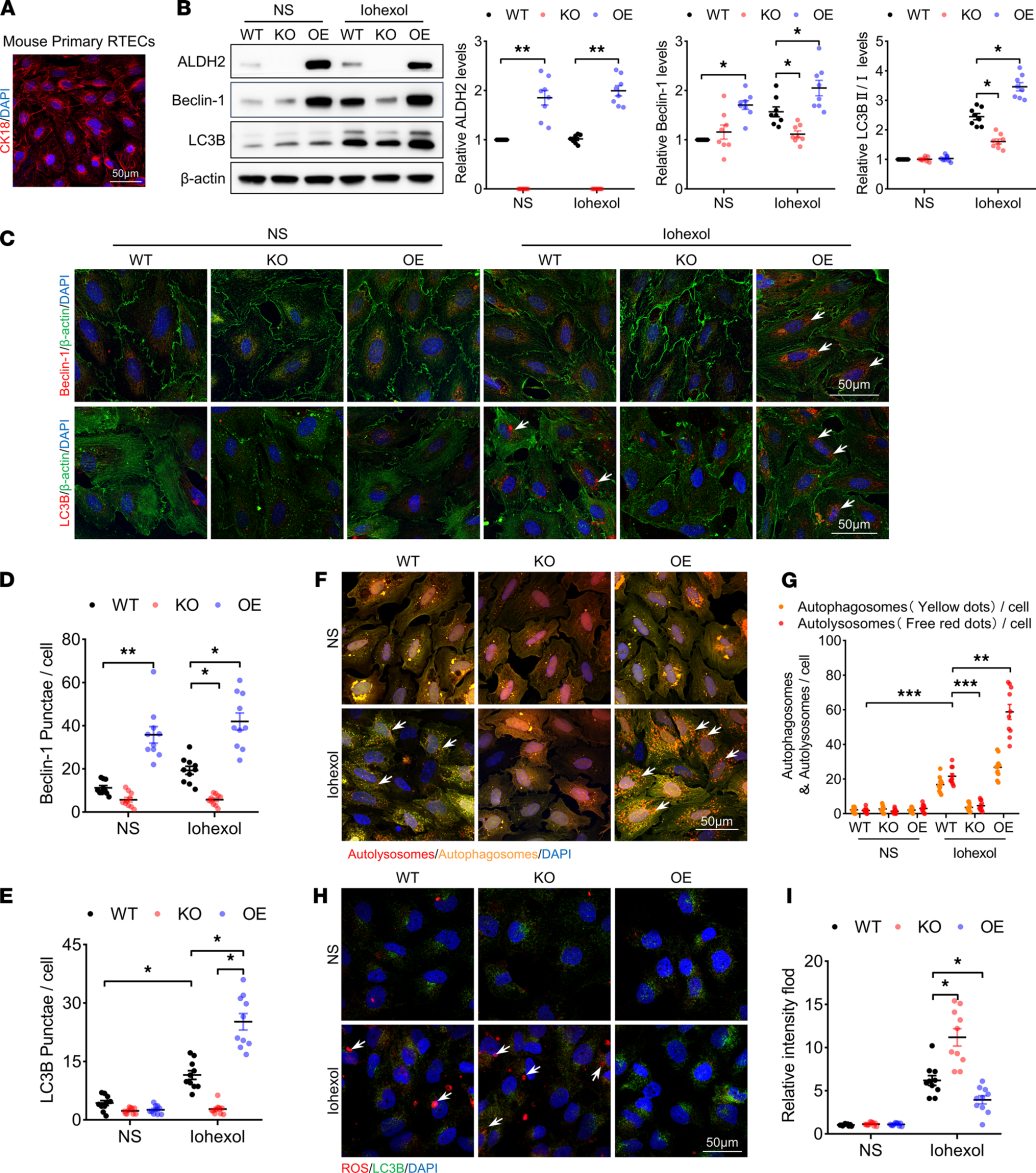
ALDH2 OE promoted autophagy activation in RTECs. (**A**) Representative images of immunofluorescence staining of cytokeratin 18 (CK18) in primary RTECs. Scale bar: 50 μm. (**B**) Immunoblotting analysis and quantification of ALDH2, Beclin-1, and LC3B in primary RTECs. (**C**–**E**) Representative images and quantification of immunofluorescence staining of Beclin-1 and LC3B in primary RTECs. Scale bar: 50 μm. (**F** and **G**) Representative images and quantification of autophagic flux in primary RTECs transfected with Ad-RFP-GFP-LC3. Scale bar: 50 μm. (**H** and **I**) Representative images and quantification of staining of ROS in primary RTECs. Scale bar: 50 μm. Data are shown as the mean ± SEM. Statistical analyses were performed using 1-way ANOVA with a post hoc test (**B**, **D**, **E**, **G**, and **I**). *n =* 8–10. *P < 0.05, **P < 0.01, ***P < 0.001.

Previous studies had shown that autophagy activation in RETCs is crucial to ALDH2-mediated renoprotection. To identify the mechanism of ALDH2-induced autophagy activation in RTECs, we isolated primary RTECs and identified cell types using immunofluorescence analysis of cytokeratin 18 (CK18, a mature epithelial cell marker; Figure 5A).

After iohexol treatment, RTECs from *ALDH2*-OE mice displayed autophagy activation compared with WT mice, as evinced by dramatic increases in Beclin-1 level and LC3BII/LC3BI ratio (Figure 5B). Likewise, immunofluorescence confocal microscopy analyses showed a marked increase in Beclin-1 and LC3B protein levels in RTECs from ALDH2-OE mice (Figure 5, C–E). Furthermore, the numbers of both autophagosomes (yellow dots) and autolysosomes (free red dots) were higher in RTECs from ALDH2-OE mice (Figure 5, F and G). However, ALDH2 deficiency abolished autophagy activation with significantly lower Beclin-1 and LC3BII protein levels and impaired autophagosome formation (Figure 5, C–G). To further explore the role of ALDH2 in sensing intracellular ROS changes and initiating autophagy, we measured the level of intracellular ROS in RTECs. ROS production was significantly increased in ALDH2-deficient RTECs under iohexol treatment, although it was markedly decreased in ALDH2-OE RTECs (Figure 5, H and I).

### ALDH2 activation promoted autophagy activation in RTECs.

**Figure 6:**
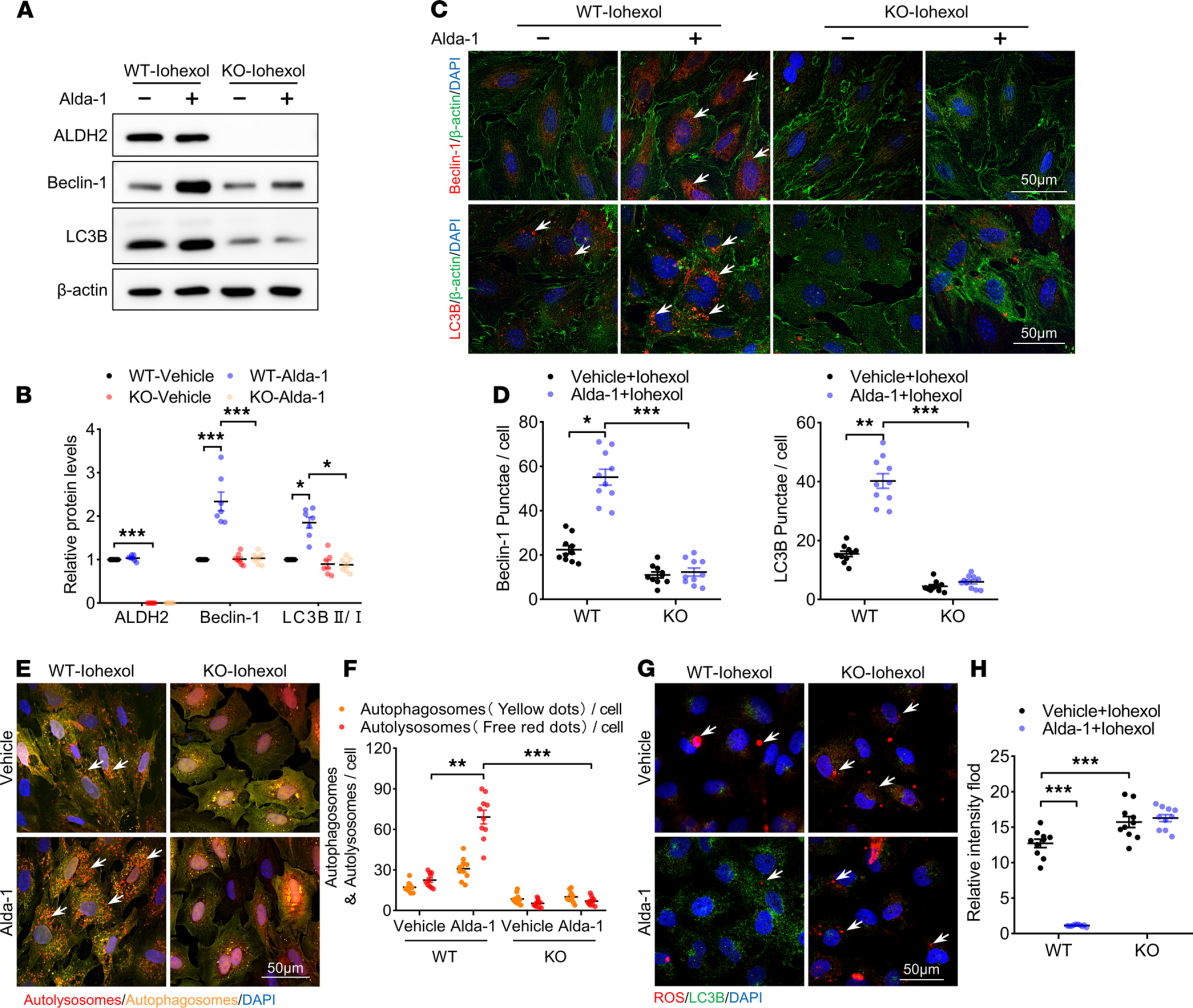
ALDH2 activation promoted autophagy activation in RTECs. (**A** and **B**) Immunoblotting analysis and quantification of ALDH2, Beclin-1, and LC3B in primary RTECs. (**C** and **D**) Representative images and quantification of immunofluorescence staining of Beclin-1 and LC3B in primary RTECs. Scale bar: 50 μm. (**E** and **F**) Representative images and quantification of autophagic flux in primary RTECs transfected with Ad-RFP-GFP-LC3. Scale bar: 50 μm. (**G** and **H**) Representative images and quantification of staining of ROS in primary RTECs. Scale bar: 50 μm. Data are shown as the mean ± SEM. Statistical analyses were performed using 1-way ANOVA with a post hoc test (**B**, **D**, **F**, and **H**). *n =* 7–10. *P < 0.05, **P < 0.01, ***P < 0.001.

Next, we investigated the role of ALDH2 in autophagy activation using Alda-1, a ALDH2-specific agonist. Alda-1 pretreatment increased Beclin-1 protein level and LC3BII/LC3BI ratio in iohexol treatment RTECs (Figure 6, A and B). Immunofluorescence confocal microscopy analyses also showed that Alda-1 pretreatment increased the protein levels of Beclin-1 and LC3B as well as the numbers of both autophagosomes and autolysosomes (Figure 6, C–F). However, Alda-1 pretreatment-induced autophagy activation, as evidenced by the elevations of Beclin-1 and LC3BII protein levels, was diminished in ALDH2-deficient RTECs. Compared with WT, Alda-1 pretreatment scarcely reversed the *ALDH2*-KO –induced reduction in Beclin-1 level, LC3II/LC3I ratio, and formation of autophagosomes and autolysosomes (Figure 6, A–F). Moreover, Alda-1 pretreatment significantly decreased ROS accumulation in WT RTECs after iohexol treatment. However, ALDH2 deficiency abolished the effect of Alda-1 limiting ROS levels (Figure 6, G and H).

### Beclin-1 plays a critical role in ALDH2-mediated autophagy in RTECs.

**Figure 7:**
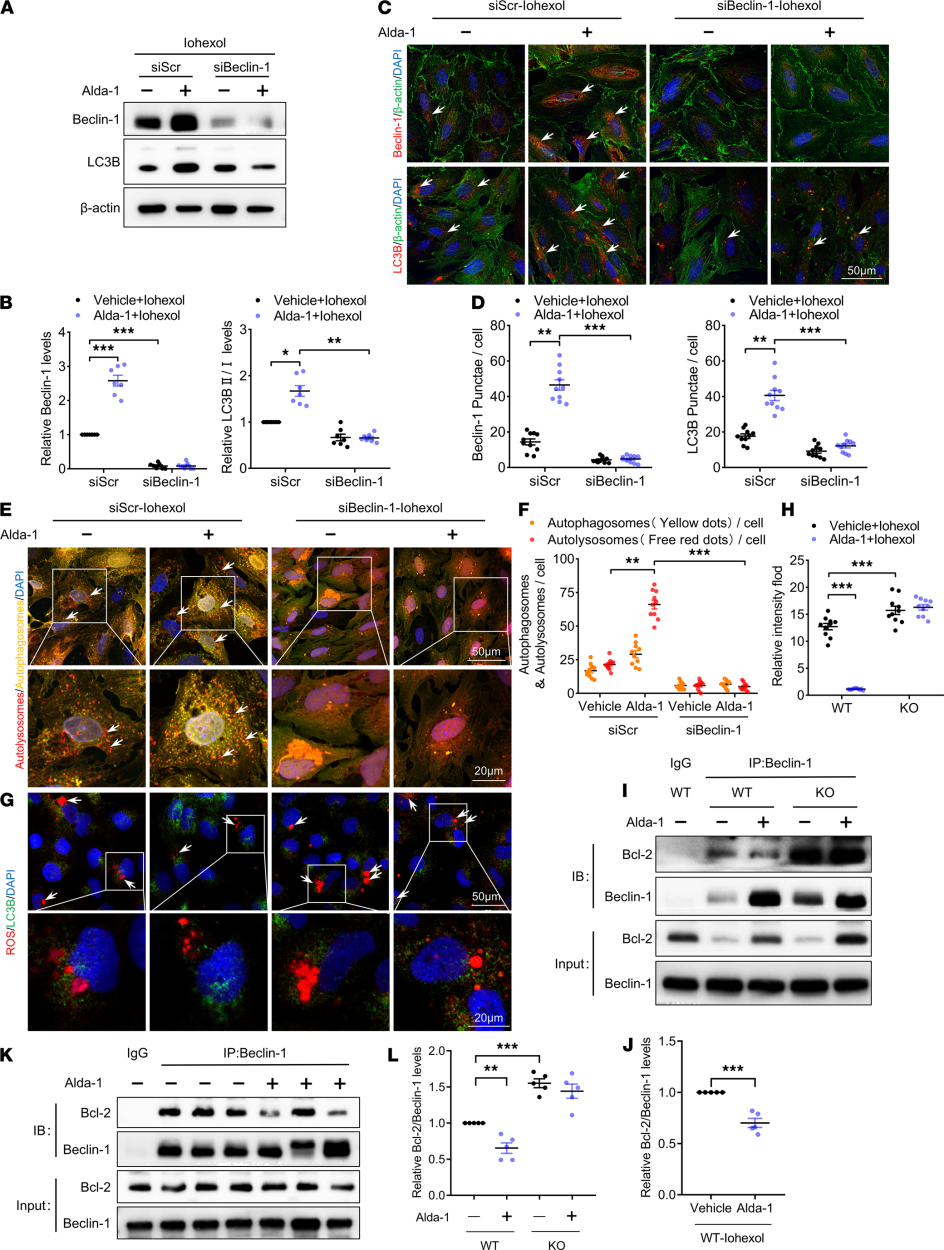
Beclin-1 plays a critical role in ALDH2-mediated autophagy in RTECs. (**A** and **B**) Immunoblotting analysis and quantification of ALDH2, Beclin-1, and LC3B in primary WT RTECs. (**C** and **D**) Representative images and quantification of immunofluorescence staining of Beclin-1 and LC3B in primary WT RTECs. Scale bar: 50 μm. (**E** and **F**) Representative images and quantification of autophagic flux in primary WT RTECs transfected with Ad-RFP-GFP-LC3. Scale bar: 50 μm (top), 20 μm (bottom). (**G** and **H**) Representative images and quantification of staining of ROS in primary WT RTECs. Scale bar: 50 μm (top), 20 μm (bottom). (**I**–**L**) IP analysis and quantification of the physical interaction between Bcl-2 and Beclin-1 proteins in RTECs. Lysates were extracted for IP with Beclin-1–specific antibody or control IgG, followed by probing with antibodies specific for Bcl-2. Data are shown as the mean ± SEM. Statistical analyses were performed using 1-way ANOVA with a post hoc test (**B**, **D**, **F**, **H**, and **L**) or 2-tailed unpaired Student’s *t* test (**J**). *n =* 5–10. *P < 0.05, **P < 0.01, ***P < 0.001.

Our data showed that ALDH2 upregulated Beclin-1 and autophagy activation expression. To further investigate the role of Beclin-1 in ALDH2- mediated autophagy activation, we measured the levels and subcellular distribution of autophagy-related molecules and ROS in Beclin-1 depleted and control cells in the absence or presence of Alda-1. Beclin-1 was stably silenced with siRNA in RTECs (Figure 7, A and B). Alda-1 induced autophagy in siScr transfected WT RTECs. In contrast, Alda-1–mediated autophagy activation was significantly suppressed in WT RTECs that had been transfected with siBeclin-1 (Figure 7, A–D). Furthermore, autophagosome and autolysosome formation associated with ALDH2 activation was repressed by siBeclin-1 (Figure 7, E and F). Similarly, Beclin-1 silencing abolished the effect of Alda-1 limiting ROS levels (Figure 7, G and H).

Previous studies have documented that Beclin-1 interacts with Bcl-2 via the BH3 domain. If the 2 proteins disassociate from each other, Bcl-2 loses its inhibitory function on Beclin-1–mediated autophagy. Our data showed that ALDH2 activation decreased the association between Bcl-2 and Beclin-1 (Figure 7, I and J). Consistently, ALDH2 deficiency inhibited Beclin-1 release from Bcl-2 sequestration (Figure 7, K and L). To elucidate how ALDH2 regulated autophagy, we investigated whether ALDH2 directly interacts with Beclin-1. IP analysis revealed that Beclin-1 interacts with ALDH2, which was strengthened by Alda-1 pretreatment (Supplemental Figure 4). Moreover, enhanced phosphorylation of Beclin-1 at Ser90 was detected in ALDH2-activated RTECs, which was exactly located within the Beclin-1–binding sequence in Bcl-2 (Supplemental Figure 4). Taken together, these results clearly confirmed that Beclin-1 is necessary for ALDH2-mediated autophagy activation.

### ALDH2 knockdown in RTECs impaired autophagy activation and aggravated renal injury in CI-AKI mice.

**Figure 8:**
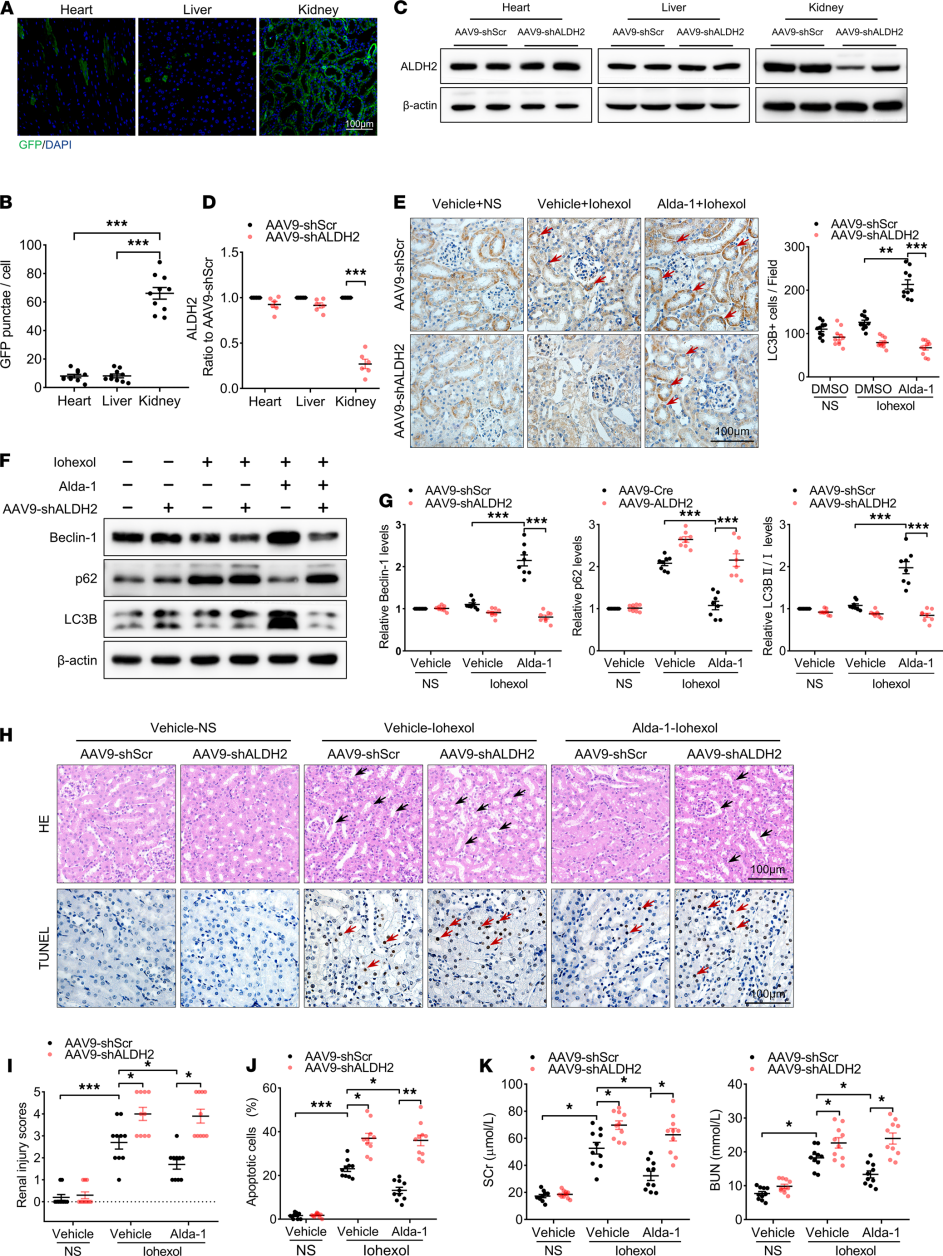
ALDH2 knockdown in RTECs impaired autophagy activation and aggravated renal injury in CI-AKI mice. (**A** and **B**) Representative images and quantification of immunofluorescence staining of GFP in the renal cortex from AAV9-Ksp-GFP-shALDH2 injection mice. Scale bar: 50 μm. (**C** and **D**) Immunoblotting analysis and quantification of ALDH2 in the primary renal cortex. (**E**) Representative images and quantification of immunohistochemical staining of LC3B in the renal cortex. LC3B-positive cells are indicated by red arrows. Scale bar: 100 μm. (**F** and **G**) Immunoblotting analysis and quantification of Beclin-1, p62, and LC3B in the renal cortex. (**H**–**J**) Representative images and quantification of TUNEL staining in the renal cortex. TUNEL-positive cells are indicated by red arrows. Scale bar: 100 μm. (**K**) Renal function was evaluated by SCr and BUN. Data are shown as the mean ± SEM. Statistical analyses were performed using 1-way ANOVA with a post hoc test (**B**, **D**, **E**, **G**, **J**, and **K**) or χ^2^ test (**I**). *n =* 10. *P < 0.05, **P < 0.01, ***P < 0.001.

C57BL/6J mice were injected via the tail vein with AAV9-Ksp-GFP-shALDH2 (AAV9-shALDH2) or the control vector AAV9-Ksp-GFP-shScramble (AAV9-shScr). Two weeks after injection, these mice were used to establish the CI-AKI model. As shown in Figure 8, A–D, 2 weeks after tail vein injection, AAV9-shALDH2 specifically infected RTECs and significantly decreased the protein levels of ALDH2 in the renal cortex, indicating that AAV9-shALDH2–induced ALDH2 knockdown was specific to RTECs. Further immunohistochemical staining revealed that Alda-1 pretreatment increased LC3B protein levels in RTECs in AAV9-shScr CI-AKI mice. However, Alda-1 induction of LC3B was attenuated in RTECs of AAV9-shALDH2–injected CI-AKI mice (Figure 8E). Similarly, Alda-1 pretreatment mediated autophagy activation in the renal cortex of AAV9-shScr–injected CI-AKI mice, as demonstrated by higher Beclin-1 levels and LC3BII/LC3BI ratio. and lower p62 protein levels. However, Alda-1–induced autophagy was inhibited in the renal cortex of AAV9-shALDH2–injected CI-AKI mice (Figure 8, F and G). Collectively, RTECs were involved in ALDH2-mediated autophagy in CI-AKI.

To further clarify the role and regulation of ALDH2-mediated autophagy in CI-AKI, we assessed microscopic pathology of the kidney cortex with H&E staining and TUNEL staining. H&E staining revealed that the renal cortex of AAV9-Scr– or AAV9-shALDH2–injected CI-AKI mice exhibited intraepithelial vacuolar degeneration, tubular dilation, and apoptosis of RTECs. Compared with AAV9-shScr injection, the renal tubular injury score and apoptosis of the AAV9-shALDH2 injection CI-AKI mice were higher. Next, Alda-1 pretreatment attenuated renal injury and apoptosis in AAV9-shScr–injected CI-AKI mice, whereas protection by Alda-1 was abolished in AAV9-shALDH2–injected CI-AKI mice (Figure 8, H–J). Moreover, compared with those receiving a control AAV9-shScr injection, the SCr and BUN levels were significantly increased in AAV9-shALDH2–injected CI-AKI mice regardless of whether they had been pretreated or not with Alda-1 (Figure 8K). These data demonstrated that ALDH2-mediated autophagy in RTECs was activated to prevent renal injury in CI-AKI.

### ALDH2 activation mediated autophagy activation and attenuated apoptosis in HK-2 cells exposed to iohexol.

**Figure 9:**
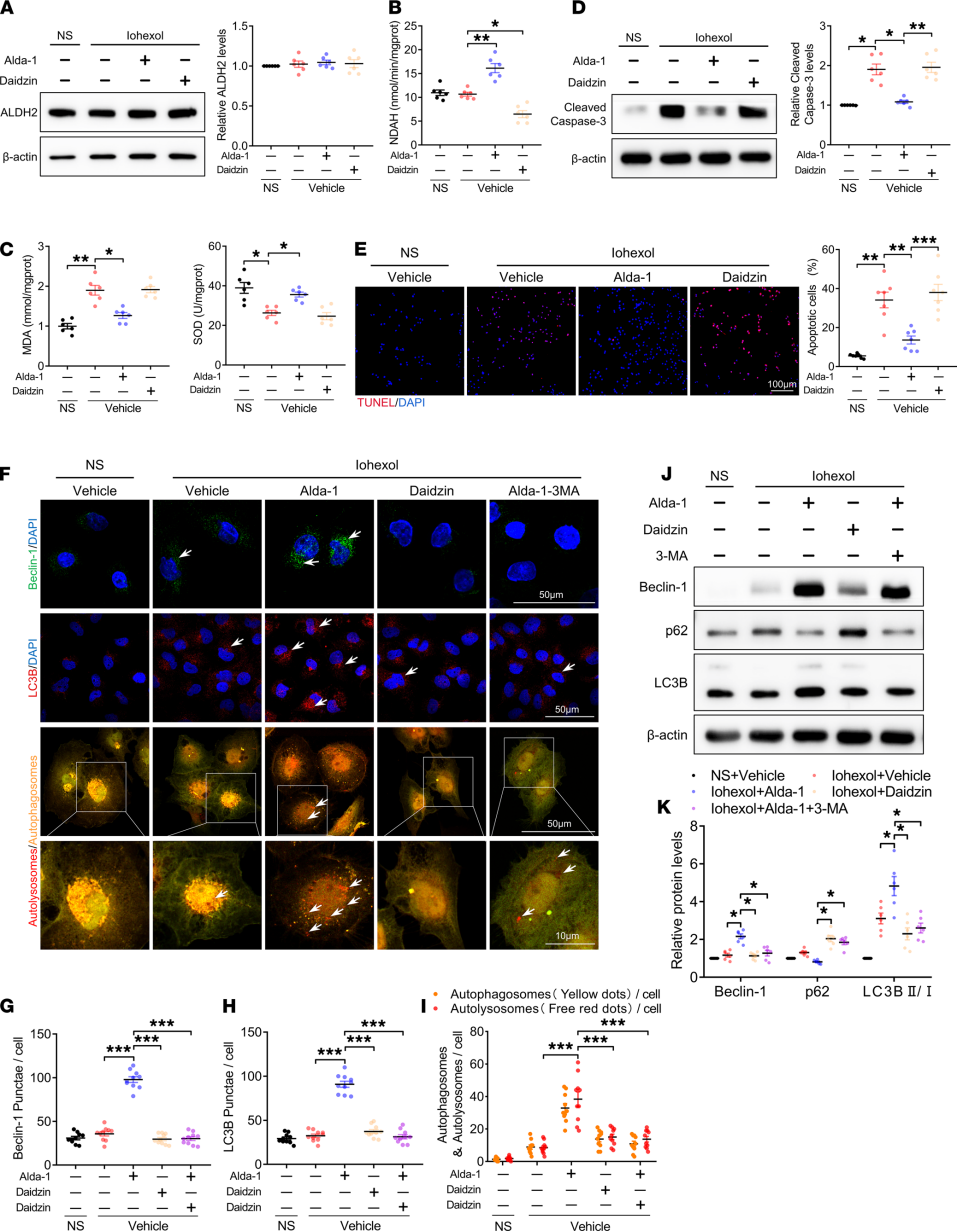
ALDH2 activation mediated autophagy activation and attenuated apoptosis in HK-2 cells exposed to iohexol. (**A**) Immunoblotting analysis and quantification of ALDH2 in HK2 cells. (**B**) ALDH2 enzymatic activity in HK-2 cells. (**C**) MDA and SOD expression in HK-2 cells. (**D**) Immunoblotting analysis and quantification of cleaved caspase-3 in HK-2 cells. (**E**) Representative images and quantification of TUNEL staining in HK-2 cells. Scale bar: 100 μm. (**F**–**I**) Representative images and quantification of immunofluorescence staining of Beclin-1, LC3B, and autophagic flux in HK-2 cells. Scale bar: 50 μm (first, second, and third row), 10 μm (bottom row). (**J** and **K**) Immunoblotting analysis and quantification of Beclin-1, p62, and LC3B in HK-2 cells. Data are shown as the mean ± SEM. Statistical analyses were performed using 1-way ANOVA with a post hoc test (**A**–**K**). *n =* 6–10. *P < 0.05, **P < 0.01, ***P < 0.001.

We investigated the specific function of ALDH2 in human renal proximal tubular epithelial cell lines (HK-2 cells). Immunoblotting revealed that iohexol did not significantly change ALDH2 protein levels in HK-2 cells. Additionally, Alda-1 significantly enhanced ALDH2 activity in HK-2 cells, whereas daidzin had the opposite effect, with neither significantly affecting ALDH2 expression (Figure 9, A and B). As shown in Figure 9C, iohexol treatment increased MDA level and decreased SOD level in HK-2 cells, whereas Alda-1 pretreatment markedly attenuated these changes. Apoptosis was examined in HK-2 cells using immunoblotting and TUNEL staining. Alda-1 pretreatment mitigated the iohexol-induced enhanced levels of cleaved caspase-3 and the number of TUNEL-positive cells (Figure 9, D and E). Moreover, immunofluorescence confocal microscopy analyses revealed that Alda-1 pretreatment markedly increased Beclin-1 and LC3B protein levels as well as the formation of autophagosomes and autolysosomes in iohexol-induced HK-2 cells (Figure 9, F–I). Immunoblotting also demonstrated that Alda-1 pretreatment dramatically increased Beclin-1 level and LC3BII/LC3BI ratio in iohexol-induced HK-2 cells. However, 3-MA largely abolished ALDH2-mediated autophagy in HK2 cells (Figure 9, J and K).

### ALDH2 regulated autophagy and protected against renal IRI.

**Figure 10:**
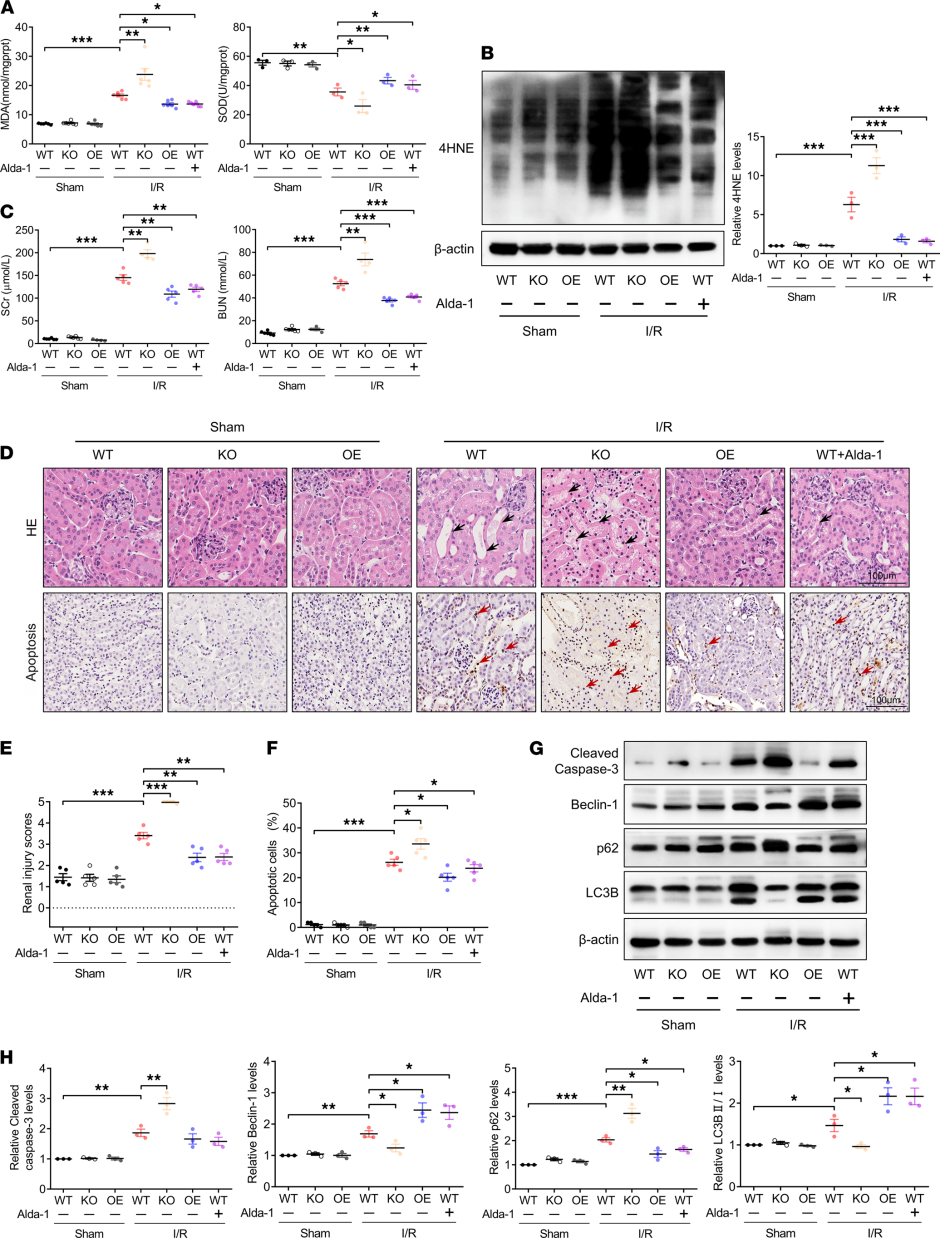
ALDH2 regulated autophagy and protected against renal ischemia/reperfusion injury. (**A**) MDA and SOD expression in the renal cortex. (**B**) Immunoblotting analysis and quantification of 4HNE in the renal cortex. (**C**) Renal function was evaluated by SCr and BUN. (**D**–**F**) Representative images and quantification of H&E staining and TUNEL staining in the renal cortex. TUNEL-positive cells are indicated by red arrows. Scale bar: 100 μm. (**G** and **H**) Immunoblotting analysis and quantification of Beclin-1, p62, and LC3B in the renal cortex. Data are shown as the mean ± SEM. Statistical analyses were performed using 1-way ANOVA with a post hoc test (**A**–**C**, **F**, and **H**) or χ^2^ test (**E**). *n =* 3–5. *P < 0.05, **P < 0.01, ***P < 0.001.

Finally, to confirm the broad implications of ALDH2 in conferring renal protection after AKI, we sought to investigate whether ALDH2 also plays a critical role in ameliorating renal ischemia/reperfusion-induced AKI. It was found that renal ischemia/reperfusion increased MDA and 4HNE accumulation in the renal cortex. Compared with WT mice, renal ischemia/reperfusion-induced ROS in the renal cortex was markedly increased in *ALDH2-*KO mice, which was significantly attenuated in ALDH2-OE or ALDH2 pretreatment mice (Figure 10, A and B). Likewise, renal ischemia/reperfusion-induced renal dysfunction was aggravated in *ALDH2-*KO mice, with a marked increase of SCr and BUN, whereas ALDH2 OE or Alda-1 pretreatment mitigated renal ischemia/reperfusion-induced renal dysfunction (Figure 10C). H&E and immunohistochemical staining revealed that renal ischemia/reperfusion-induced renal tubular injury score, and number of TUNEL-positive cells were significantly increased in *ALDH2-*KO mice, which was attenuated in ALDH2-OE or Alda-1 pretreatment mice (Figure 10, D–F). Consistently, immunoblotting analysis revealed that renal ischemia/reperfusion upregulated the cleaved caspase-3 expression in the renal cortex. Compared with WT renal IRI mice, the cleaved caspase-3 levels were higher in the *ALDH2-*KO renal IRI mice and lower in *ALDH2*-OE or Alda-1 pretreatment renal IRI mice (Figure 10, G and H). Additionally, Beclin-1 level and LC3BII/LC3BI ratio were lower in *ALDH2-*KO renal IRI mice and higher in *ALDH2*-OE or Alda-1 pretreatment renal IRI mice (Figure 10, G and H).

## Discussion

The function and regulation of ALDH2 in acute or CKD remain unknown. In this study, we first showed that ALDH2 deficiency increases ROS production, 4HNE accumulation, and DNA oxidative damage, contributing to RTECs apoptosis and tubular damage in CI-AKI. We next demonstrated that ALDH2 activation protects against CI-AKI by decreasing ROS and cellular apoptosis. Previous studies indicated that ROS was one of the factors involved in AKI pathogenesis. The excessive generation of ROS induced by contrast agent exposure directly causes tubular damage, endothelial dysfunction, and dysfunction of tubular transport. The imbalance in reactive species causes lipid peroxidation accompanied by production of MDA and 4HNE, leading to cytotoxic damage.

Furthermore, we aimed to identify and examine ALDH2-mediated mechanisms in protecting against CI-AKI. Here, we present genome-wide transcript data from the renal cortex samples from CI-AKI mice. According to the results of RNA-Seq, we demonstrated that genes associated with autophagy were upregulated in Alda-1 (a selective ALDH2 activator) pretreated CI-AKI mice. Subsequent data localized the results of RNA-Seq and the role of ALDH2 in autophagy to RTECs. Additionally, renoprotection by ALDH2 was abolished by the autophagy inhibitor 3-MA. These findings indicated a potentially novel correlation between ALDH2, Beclin-1–mediated autophagy, and renal injury in CI-AKI.

Consistent with the results of RNA-Seq, we found that there was a direct correlation in ALDH2 and Beclin-1 expression in primary RTECs. Further studies demonstrated that ALDH2 activation also promoted Beclin-1 expression. Silencing Beclin-1 by siRNA inhibited ALDH2-mediated autophagy and RTECs protection by decreasing LC3BII protein levels and autophagosomes formation while increasing ROS levels. These observations suggest that Beclin-1 is an essential mediator of the renoprotective effects of ALDH2-mediated autophagy in response to contrast agent.

Beclin-1 is an adaptor protein that assembles into a core complex with PI3KC3/VPS34 (vacuolar sorting protein-34) and functions in autophagy initiation (50). Additionally, Beclin-1 can also be involved in autophagic suppression of apoptosis. Beclin-1 is a multi-domain protein with an N-terminal Bcl-2 homology BH3 domain (51). The interaction between Beclin-1 BH3 domain and Bcl-2/BCL-XL stabilizes Beclin-1 dimers, effectively suppressing autophagosome biogenesis. Upon cell stress, Beclin-1 dissociates from Bcl-2 to initiate the formation of the PI3KC3 (class III phosphatidylinositol 3-kinase) complex for autophagy. Thus, the activation of autophagy may release Bcl-2 (from Beclin-1), an important anti-apoptotic protein, to protect cells from death (50).

Beyond upregulating Beclin-1 expression, our results show that ALDH2 activation disrupted the interaction between Beclin-1 and Bcl-2, thereby promoting autophagy. Moreover, ALDH2 activation enhanced phosphorylation of Beclin-1 at Ser90 within the Bcl-2 binding domain of Beclin-1 (52, 53). These results are consistent with the previous observations that the phosphorylation of Beclin-1 at Ser90 positively regulates autophagy. It should be noted that the basis for how ALDH2 enhances Beclin-1 phosphorylation triggers autophagy remains unknown, but could relate to the interaction between ALDH2 and Beclin-1.

ALDH2, a mitochondrial enzyme, is known for its detoxifying properties, which provide living organisms with a protective shield against endogenous and exogenous toxic agents, such as acetaldehyde (alcohol metabolism) and products originating from lipid peroxidation (4HNE, MDA) and ROS (54, 55). The relevance of ALDH2 in providing strong protection toward toxic damage has been described in numerous reports, demonstrating its efficacy in various models of human diseases such as ischemia/reperfusion and ischemic stroke characterized by overwhelming oxidative stress (31, 45). However, most of these studies focused on the direct detoxification effect of ALDH2 and ignored its interactions with other molecules regulating cell fate such as those involved in autophagy. Autophagy maintains cell and tissue homeostasis by suppressing ROS and clearing damaged organelles (53). Specifically, our results indicate that removal of ROS via Beclin-1–induced autophagy is an important mechanism underlying renoprotection by ALDH2.

Subsequent studies in HK-2 cells and IRI mice confirmed the potential renoprotective effect of ALDH2 in multiple forms of AKI. In the present study, Alda-1 infusion prior to ischemia reduced IRI-induced renal damage, which suggests that renal expression levels of ALDH2 play a critical role in regulating the pathological changes of IRI-induced renal injury. Although in a previous study, a longer period of Alda-1 infusion during the renal after ischemia period resulted in intratubular crystal formation and deterioration of renal function (47) and several factors, such as the degree of drug supersaturation and underlying kidney diseases, can lead to the intratubular deposition of crystals (56–58), in other studies, much longer Alda-1 infusion in various nonrenal conditions did not lead to crystal formation, indicating that renal ischemia is the key factor in crystalline nephropathy (59, 60). Therefore, Alda-1 should be used with caution in these conditions or a safer and more effective ALDH2 activator should be used.

More importantly, our study emphasized that the *ALDH2* gene mutation may also be a risk factor for CI-AKI and renal IRI. Patients undergoing percutaneous coronary intervention or coronary angiography are most likely to be exposed to large doses of contrast agents at one time. Therefore, patients with coronary heart disease and *ALDH2* gene mutation might to be at greater risk of CI-AKI and renal IRI. Further clinical studies are warranted to fully unveil the protective properties and clinical implications of ALDH2 in AKI, and to examine the association between the *ALDH2* gene and AKI risk. The observations in the present study are relevant to the diagnosis and treatment of AKI in patients with *ALDH2* Lys504 mutation (present in 30%–50% of East Asians, and in 8% of the global population).

In summary, we demonstrated in vivo and in vitro that ALDH2 protects RTECs from the untoward effects of contrast agent exposure and renal ischemia/reperfusion. ALDH2 via the Beclin-1–autophagy axis prevents RTEC apoptosis and tissue damage by cleaning ROS and improves survival of RTECs in CI-AKI and renal IRI. Therapeutic strategies aimed at activation of ALDH2 may therefore preserve tubular epithelial cells against CI-AKI.

## Methods

### Animals.

*ALDH2-*KO and -OE mice were obtained from the Riken Bioresource Center on a C57BL/6J background of the same strain and backcrossed for at least 10 generations with in-house C57BL/6J mice to create a congenic strain. After backcross, *ALDH2-*KO and -OE mice heterozygotes on C57BL/6J genetic background were mated separately to generate control and experimental groups of mice. Mice were genotyped by PCR analyses of genomic DNA isolated from mouse tails; homozygous ALDH2-KO and -OE mice were used in experimental groups and WT littermate mice were used as controls. The mice genotyping data are provided in Supplemental Figure 1, C and D. All mice were housed in the experimental animal center of Qilu Hospital of Shandong University.

### CI-AKI and IRI models.

Eight-to-10-week-old adult male WT, *ALDH2-*KO, and-OE mice were used for the CI-AKI model. After overnight (16 hours) water deprivation and inhibition of prostaglandin and nitric oxide synthesis, mice were injected i.p. with the low-osmolar monomeric iodinated radiocontrast medium iohexol (4 mg/g). For inhibition of cyclooxygenase and nitric oxide synthesis, mice were injected i.p. with indomethacin (I106885, Aladdin; 10 mg/kg, dissolved in DMSO) and N-nitro-l-arginine methyl ester (L-NAME; S2877, Selleckchem, 10 mg/kg, dissolved in 0.9% saline). Iohexol was injected i.p. 15 minutes later for the CI-AKI model, whereas the same volume of physiological saline was used for the control. Then, animals were given water and food. Twenty-four hours later, after isoflurane (2%) inhalation and cervical dislocation, kidneys and blood were collected for laboratory assessments. Before the CI-AKI mouse models, Alda-1 (20 mg/kg), daidzin (75 mg/kg), rapamycin (2 mg/kg), or 3-methyladenine (15 mg/kg) were i.p. injected for 3 days in different groups. The latter model was validated to reliably produce nephropathy following radiocontrast injection in mice and rats (61, 62).

In the IRI model, after isoflurane (2%) inhalation, mice (7–8 weeks old, males) were placed on a heating pad to maintain core body temperature at 37°C, and the body was covered with gauze soaked in warm water to preserve moisture. Both kidney pedicles were identified through 2 small paramedial dorsal incisions and clamped for 28 minutes. Control animals underwent the same surgical procedure without having their kidney pedicles clamped. After isoflurane (2%) inhalation and cervical dislocation, kidneys and blood samples were collected at 24 hours after clamping and reperfusion. Before the IRI models, Alda-1 (20 mg/kg) was i.p. injected for 3 days in the Alda-1–treated group.

### Isolation and cell culture of primary mouse RTECs.

Primary mouse RTECs were obtained and cultured as previously described (63). Briefly, male mice aged 4–6 weeks were anesthetized and flushed with 5 mL ice-cold HBSS (Gibco, 14175095). Renal cortex was dissected visually and sliced into small pieces. The fragments were transferred through 2 layers of nylon sieves (pore size, 125 and 106 μm, respectively). After sieving, mouse tubular fragments were selected and seeded in collagen precoated flasks (Corning) with DMEM/F12 (Gibco, 11039-021), in the presence of 10% heat-inactivated FBS (Gibco, 10099-147), 1% L-glutamine, and 1% penicillin/streptomycin. The plate was incubated in a standard humidified incubator equipped with 5% CO_2_. The medium was changed 2 days later and maintained every other day until the monolayer of cells reached 90% confluence. Immunostaining against CK18 (Abcam, 133263) confirmed the presence of tubular cells. RTECs were treated with Alda-1 (20 μmol) or vehicle for 1 hour at 37°C before 4 hours exposure to the low-osmolar iodinated radiocontrast agent iohexol (6 mmol/L).

HK-2 cells (ATCC) were grown and passaged in DMEM/F12 with 10% FBS (HyClone) and antibiotics (100 U/mL penicillin G, 100 mg/mL streptomycin, and 0.25 mg/mL amphotericin B [Invitrogen]) at 37°C in a 100% humidified atmosphere of 5% CO_2_/95% air. HK-2 cells were treated with Alda-1 (20 μmol); daidzin (60 μM); rapamycin (20 nM), and 3-methyladenine (5 mM) or with vehicle for 1 hour at 37°C before 4 hours exposure to the low-osmolar iodinated radiocontrast agent iohexol (6 mmol/L).

### Measurement of ALDH2 activity.

A Cell Mitochondria Isolation Kit (C3601, Beyotime) was used to extract mitochondrial protein following the manufacturer’s protocol. ALDH2 activity then was measured in 33 mmol/L sodium pyrophosphate containing 0.8 mmol/L NAD^+^, 15 mmol/L propionaldehyde, and 0.1 mL mitochondrial protein extract. Propionaldehyde, the substrate of ALDH2, was oxidized in propionic acid, and NAD^+^ was reduced to NADH to estimate ALDH2 activity. NADH was determined by spectrophotometric absorbance at 340 nm (Microplate Reader Model Eon, BioTek). ALDH2 activity was expressed as nmol NADH/min/mg protein. Each experiment was carried out 3 times.

### Assessment of renal function.

The mice were anesthetized with 0.1% pentobarbital sodium before being sacrificed. Approximately 0.5 mL blood was collected from venae angularis. After being placed on ice for one-half an hour, blood samples were centrifuged at 3000*g* for 15 minutes to collect serum. An automatic biochemical analyzer (Cobas 8000, Hitachi) was employed to determine levels of BUN and SCr to evaluate changes in renal function.

### Morphological evaluation.

Mouse kidneys were embedded in 4% paraformaldehyde for at least 24 hours. Paraffin-embedded kidney tissue blocks were cut into 3 μm sections and subjected to H&E staining. Two pathologists blinded to this study analyzed the extent of kidney damage in these histopathological sections. Histological scoring encompassed grading of tubular necrosis, loss of brush border, cast formation, and tubular dilatation in 10 randomly chosen nonoverlapping fields. Renal injury degree was classified as: 0, none; 1, 0%–10%; 2, 11%–25%; 3, 26%–45%; 4, 46%–75%; and 5, 76%–100%.

### Measurement of apoptosis.

The Apoptosis Detection Kit (S7101, Millipore) was used for TUNEL staining to evaluate the extent of apoptosis in kidneys. HK-2 cells apoptosis was detected by TUNEL (11684795910, Roche) following the manufacturer’s instructions. After staining, paraffin sections and HK-2 cells were observed under a microscope (Olympus, X51) and analyzed using Image-Pro Plus 6.0. TUNEL-positive cells were expressed as a percentage of total cells.

### TEM.

Dissected renal cortex samples were fixed immediately in 2% glutaraldehyde and 2% paraformaldehyde in 0.1 M phosphate buffer (pH 7.4) for 2 hours at 4°C. Following 3 washes in phosphate buffer, the kidney tissues were fixed with 1% osmium tetroxide on ice for 2 hours and washed 3 times in phosphate buffer. The tissues were then embedded in Epon 812 mixture after dehydration in an ethanol and propylene oxide series. Polymerization was conducted with pure resin at 70°C for 24 hours. Ultrathin sections (approximately 70 nm) were obtained with a MT-X ultramicrotome (RMC). The sections were collected on 100 mesh copper grids. After staining with 2% uranyl acetate (15 minutes) and lead citrate (5 minutes), the sections were visualized by TEM using a Technai G2 Spirit Twin apparatus (FEI) at 120 kV.

### Measurement of oxidative stress.

To measure ROS levels, paraffin sections of kidney were incubated with 10 mm dichloro-dihydrofluorescein diacetate with a ROS assay kit (A057, Genecopoeia) at 37°C for 30 minutes in the dark. Then, sections were washed and observed under a fluorescence microscope (X51, Olympus) and analyzed using Image-Pro Plus 6.0. MDA and SOD in the kidney tissues were detected using commercially available kits. MDA contents were determined by the thiobarbituric acid method (A003-4, Nanjing Jiancheng). The SOD activity assay kit (A001-2, Nanjing Jiancheng) was used to determine SOD activity. All the measurements were made according to manufacturer’s instructions.

### mRNA-Seq.

Massively parallel mRNA-Seq was used to investigate in an unbiased fashion the expression of different genes in mouse renal cortex (duplicate samples). Twenty-four hours after establishment of the mouse CI-AKI model, kidneys were collected after isoflurane (2%) inhalation and cervical dislocation. Renal cortex was dissected quickly and preserved in liquid nitrogen. The process of sampling was very quick and all samples were kept at low temperature. After RNA isolation and purification, RNA quality was examined using the kaiaoK5500Spectrophotometer (Kaiao) and the RNA Nano 6000 Assay Kit of the Bioanalyzer 2100 system (Agilent Technologies). Sequencing libraries were generated using the NEBNext Ultra RNA Library Prep Kit for Illumina (NEB, E7530L) per the manufacturer’s instructions, and index codes were added to attribute sequences to each sample. Qubit RNA Assay Kit in Qubit 3.0 was used to check the RNA concentration of library using the Agilent Bioanalyzer 2100 system (Agilent Technologies), and qualified insert size was accurately quantified using StepOnePlus Real-Time PCR System (Library valid concentration>10 nM). The clustering of the index-coded samples was performed on a cBot cluster generation system using HiSeq PE Cluster Kit v4-cBot-HS (Illumina) following the manufacturer’s instructions. After cluster generation, the libraries were sequenced on an Illumina platform and 150 bp paired-end reads were generated (BioProject accession number PRJNA751142).

### Western blot and IP analysis.

Protein samples were separated by SDS-PAGE and transferred to PVDF membranes, which were blocked with 5% skim milk and incubated overnight at 4°C with primary anti-4HNE (46545, Abcam, 1:1000), anti-ALDH2 (108306, Abcam, 1:1000), anti-Cleaved Caspase-3 (CST9661, 1:500), anti-Beclin-1 (207612, Abcam; 62557, Abcam; 1:1000), anti-LC3B (51520, Abcam, 1:500), anti-p62 (109012, Abcam, 1:1000), anti-Phospho-Beclin-1 (Ser90) (86455S, CST, 1:1000), anti-Bcl-2 (182858, Abcam, 1:1000), and anti-β-actin (BM0005, BOSTER, 1:5000) antibodies. Then, the membranes were incubated with goat anti-mouse and goat anti-rabbit secondary antibodies (ZSGB-BIO) for 2 hours at room temperature. Finally, the membranes were scanned and detected by the chemiluminescence method. The results were normalized to β-actin protein levels.

For IP, cells were lysed with IP lysis buffer. Primary antibody was coupled with protein A/G beads (Santa Cruz), and immune complex was added to the cell lysates and incubated at 4°C overnight. After IP, the samples were washed with IP lysis buffer 3 times. Proteins were eluted with 2× SDS sample buffer.

### IHC.

Immunohistochemical assessment of muscularization was performed by staining for anti-LC3B (51520, Abcam, 1:100). Sections were photographed, and percentages of LC3B staining cells were determined.

### Immunofluorescence and confocal microscopy.

RTECs and HK2 cells were incubated with primary antibodies and levels of Beclin-1 (anti-Beclin-1, 62557, Abcam, 1:100) and LC3B (anti-LC3B, 51520, Abcam, 1:100) in cells were evaluated in images obtained using confocal laser scanning fluorescence microscopy (LSM780; Carl Zeiss AG).

### Evaluation of fluorescent LC3 puncta.

Changes in fluorescent LC3 puncta were evaluated with a tandem RFP-GFP-LC3 construct as described previously. Renal RTECs and HK-2 cells were transfected with Ad-RFP-GFP-LC3 (ViGene Biosciences Company) at 50 MOI before subsequent treatment. Green and red fluorescence intensity were assessed under a laser confocal microscope.

### siRNA and transfection.

Mouse scrambled siRNA (sc-37007, Santa Cruz Biotechnology) and siBeclin-1 (sc-29798, Santa Cruz Biotechnology) were used following the manufacturer’s instructions. Primary mouse RTECs were seeded, and 50 nmol/L scrambled siRNA or siBeclin-1 were transfected using Lipofectamine RNAiMAX per the manufacturer’s recommendations. After 48 hours of siRNA treatment, vehicle or iohexol was added for 4 hours. At the end of the experiments, cells were collected and the level of protein expression was evaluated by Western blotting.

### AAV9 production and delivery.

As previously described, 1341 bp of the 5′ flanking region (nucleotides 2430–770 of GenBank accession no. AF118228) of Ksp-cadherin (a unique, tissue-specific member of the cadherin family that is exclusively expressed in RTECs) was used as the upstream promoter of shALDH2 (64). The shScramble was prepared as a negative control sequence. GFP linked to each shRNA cassette was cloned downstream of the Ksp-cadherin promoter in the AAV9 vector plasmid. AAV9-Ksp-GFP-shALDH2 was delivered via tail vein injection into C57BL/6J mice (males, aged 4 weeks) that were used 2 weeks later to establish the CI-AKI model. ALDH2 shRNA sequence is as follows: 5′-ACTGAAATGTCTCCGCTATTACTAGTGAAGCCACAGATGTAGTAATAGCG; GAGACATTTCAGG-3′.

### Statistics.

Statistical analysis was performed using GraphPad Prism 8. All data are shown as the mean ± SEM. For continuous variables, statistical differences were determined using 2-tailed unpaired Student’s *t* test for 2-group comparisons or 1-way ANOVA with a post hoc test for multiple comparisons. For categorical variables, statistical analysis was performed using a χ^2^ test. A *P* value of less than 0.05 was considered significant.

### Study approval.

All animal procedures performed conform to guidelines from Animal Research: Reporting of In Vivo Experiments and were approved by the Animal Use and Care Committee of Shandong University.

## Author contributions

TX, FX, and YC conceived and designed the experiments. TX, JG, MW, JW, KY, LX, JZ, WS, and TJ performed the experiments. TX, MW, and JW analyzed the data. TX, FX, and YC wrote the paper. TX, JG, CP, JP, QY, and MX revised the paper for scientific content.

## Supplementary Material

Supplemental data

## Version 1 07/06/2021

In-Press Preview

## Version 2 08/09/2021

Electronic publication
