# Supplementary material for: Aldehyde dehydrogenase 2 protects against acute kidney injury by regulating autophagy via the Beclin-1 pathway
Source: JCI Insight. 2021 Aug 9;6(15):e138183. doi: 10.1172/jci.insight.138183 (PMC8410052; doi:10.1172/jci.insight.138183)
Supplement: Supplemental data [file jciinsight-6-138183-s298.pdf]

Supplemental Information

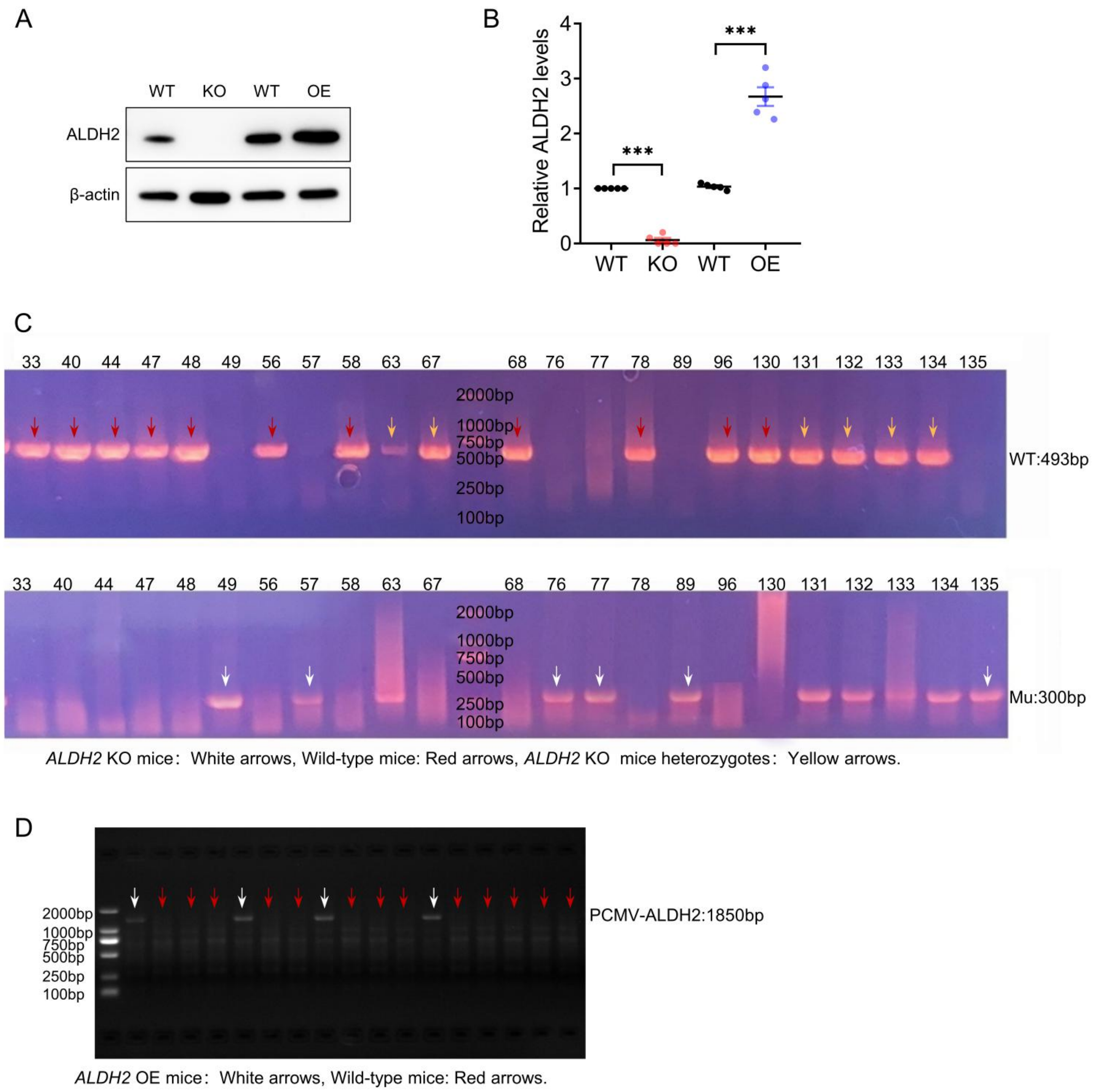

Fig S1. (A and B) Immunoblotting analysis and quantification of ALDH2 in the renal cortex. Data are presented as mean±SEM. Statistical analyses were performed using two-tailed unpaired Student's test. n=5, ***P < 0.001. (C) Representative images of PCR analyses genotyping the wild-type and *ALDH2* KO mice. The following primers were used for genotyping the wild-type and *ALDH2* KO mice, from chromosomal DNA extracted from a piece of tail. For wild-type, the primer sequences were: forward, 5'-CCGTACTGACTGTCCCATGCAGTGCT-3' and

reverse, 5'-GTGACCAGTTACAAAGGGCC-3'.

For *ALDH2* KO, the primer sequences were:

forward, 5'-CCGTACTGACTGTCCCATGCAGTGCT-3' and

reverse, 5'-GGTGGATGTGGAATGTGTGCGAGG-3'.

A PCR product of 300bp indicated *ALDH2* KO (white arrows) while 493bp band indicated wild-type (red arrows).

(D) Representative images of PCR analyses genotyping the wild-type and *ALDH2* OE mice. The following primers were used for genotyping the wild-type and *ALDH2* OE mice, from chromosomal DNA extracted from a piece of tail.

For *ALDH2* OE, the primer sequences were:

forward, 5'-GCGTGTACGGTGGGAGGTCTAT-3' and

reverse, 5'-GGCTGGCAACTAGAAGGCACAG-3'.

A PCR product of 1850bp indicated *ALDH2* OE (white arrows) while no band indicated wild-type (red arrows).

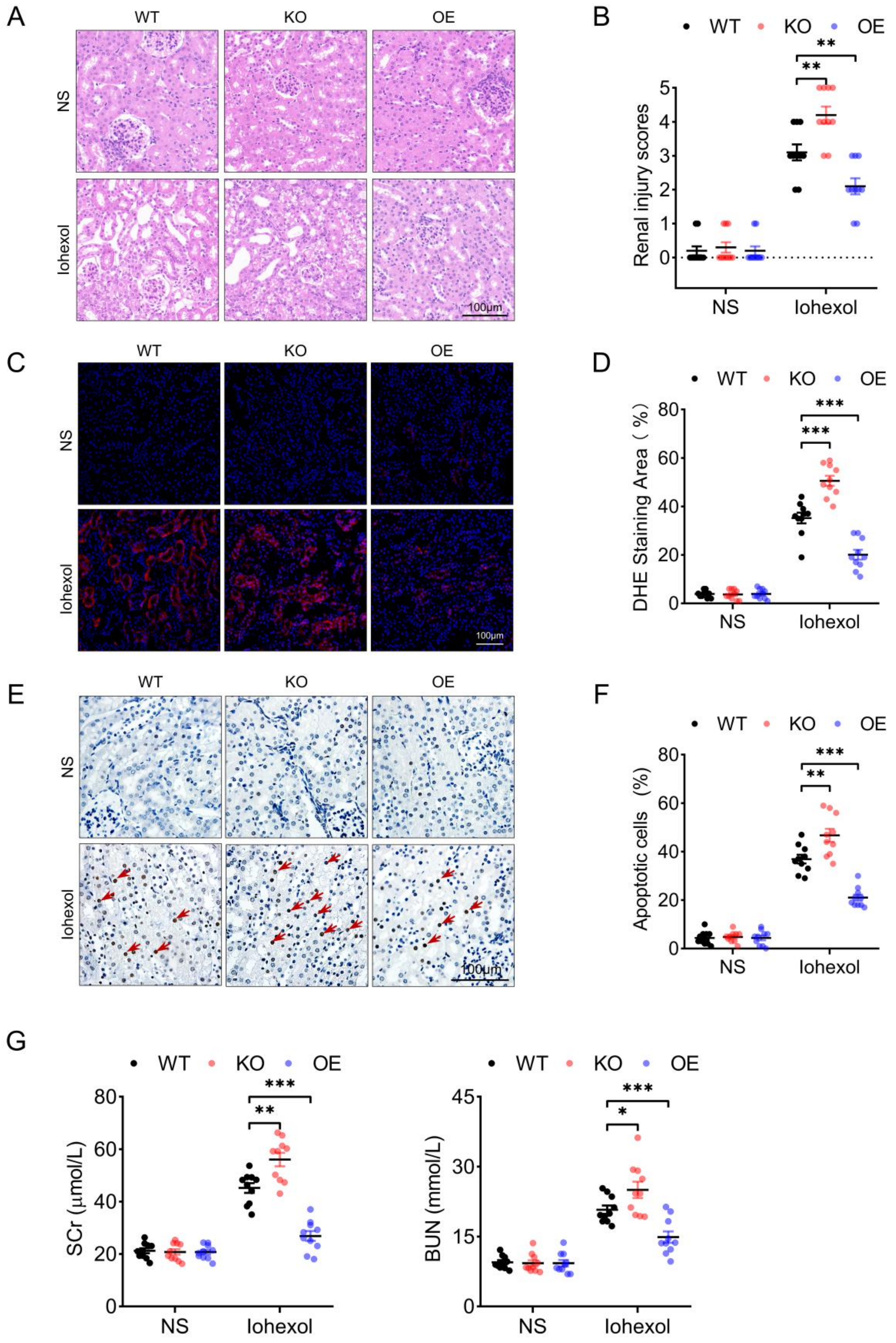

Fig S2. (A and B) Representative images and quantification of HE staining in the renal cortex. (C and D) Representative images and quantification of DHE staining (red) in the renal cortex. Scale bar: 100μm. (E and F) Representative images and quantification of TUNEL staining in the renal cortex. TUNEL positive cells are indicated by red arrows.Scale bar: 100μm. (G) Renal function was

evaluated by SCr (serum creatinine) and BUN (blood urea nitrogen). Data are presented as mean±SEM. Statistical analyses were performed using Chi square test (B) or 1-way ANOVA with a post hoc test (D, F and G). n=10. *$P< 0.05$, **$P< 0.01$, ***$P< 0.001$.

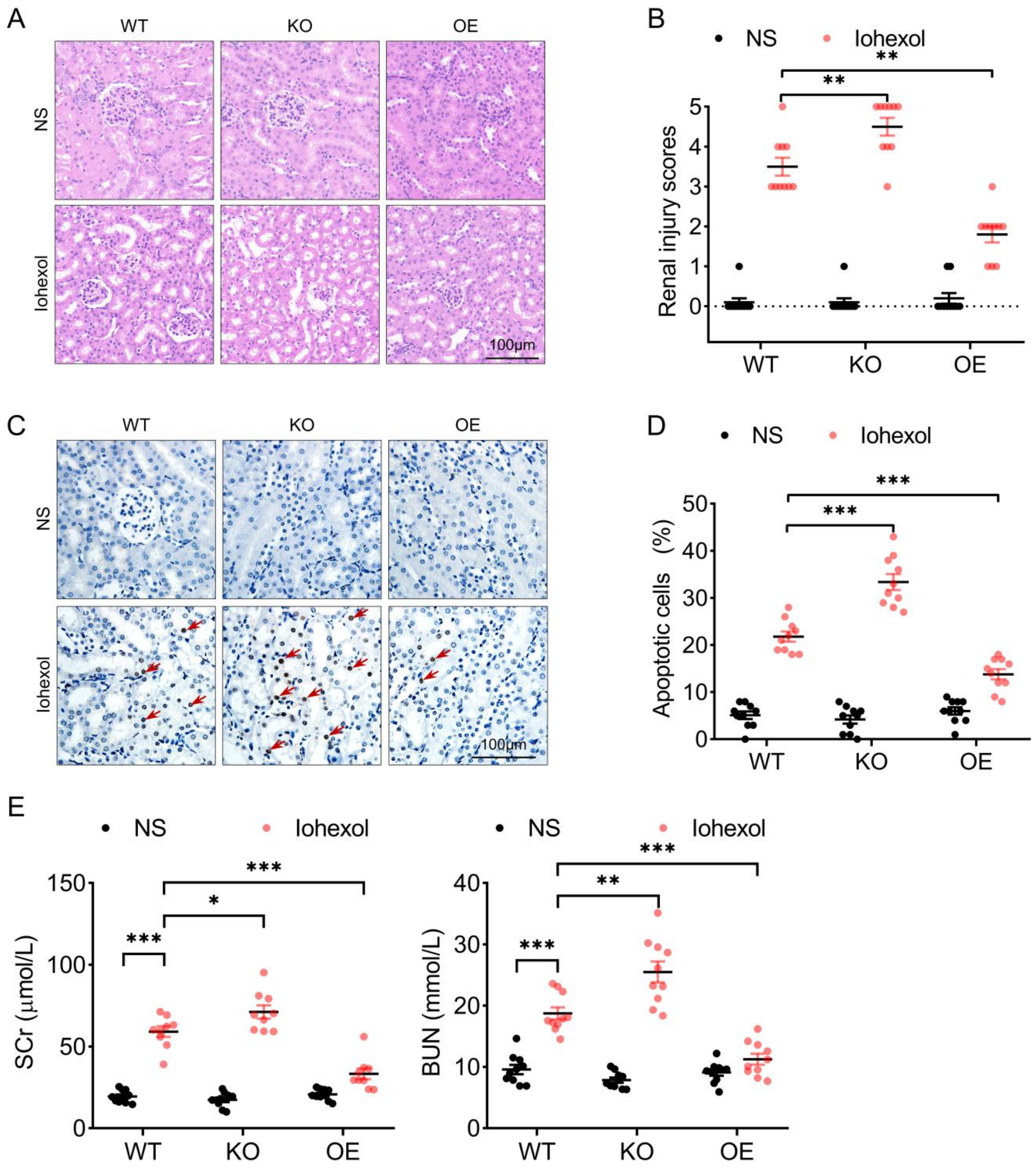

Fig S3. (A and B) Representative images and quantification of HE staining in the renal cortex. (C and D) Representative images and quantification of TUNEL staining in the renal cortex. TUNEL positive cells are indicated by red arrows.Scale bar: 100μm. (E) Renal function was evaluated by SCr (serum creatinine) and BUN (blood urea nitrogen). Data are presented as mean±SEM. Statistical analyses were performed using Chi square test (B) or 1-way ANOVA with a post hoc test (D and E). n=10. **P*< 0.05, ***P*< 0.01, ****P*< 0.001.

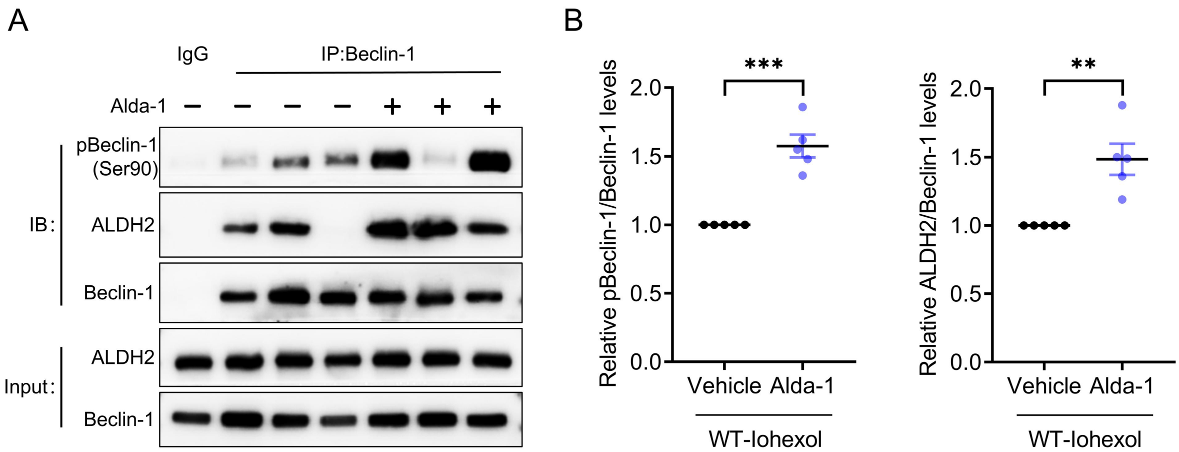

Fig S4. (A and B) Immunoprecipitation analysis and quantification of the physical interaction between pBeclin-1(Ser90), ALDH2 and Beclin-1 proteins in RTECs. Lysates were extracted for immunoprecipitation with Beclin-1-specific antibody or control IgG, followed by probing with antibodies specific for pBeclin-1(Ser90) and ALDH2. Data are presented as mean±SEM. Statistical analyses were performed using two-tailed unpaired Student's test. n=5. *$P < 0.05$, **$P < 0.01$, ***$P < 0.001$.

Table 1. List of differentially expressed genes in renal cortex from WT CI-AKI mice pretreated vs. not pretreated with Alda-1.

Gene Symbol	Log2FoldChange
Atg5	1.254037129
Becn1	1.085983873
Atg12	1.069530325
Atg10	1.349976513
Ulk1	1.022926397
Abca13	1.160435279
Hmgcll1	1.162848105
Zfp790	1.329674583
Unkl	1.17717326
Lonrf3	1.202526604
Ffar3	1.551812648
Lin7a	1.224497539
Hsp90aa1	1.040904946
Atp12a	3.088445643
Epb42	1.156824227
Sectm1a	1.318325383
Hspa5	1.015695427
Tchhl1	1.282858747
Calb1	1.219814185
Dnaja1	1.018315736
Kbtbd8	1.348764687
Tmem52b	1.138670811
Kctd7	1.035089925
Mfrp	1.55195538
Slc16a9	1.085066461
Camk4	1.067413722
Rnf146	1.129982525
Sectm1b	1.01520009
Chd3os	1.45724597
Adra1a	1.484298594
Hist1h2be	1.358180436
Vstm2a	1.372743784
Ppp1r3d	1.08367149
Zfp11	1.16155957
Casr	1.038438143
Hbb-bs	1.496066926
Lipg	2.06546018
Slc8a1	1.538368866
Ncoa4	1.067159138
Tmem178b	1.390674014

Anks1b	1.437147313
Slc10a5	1.335363296
Klk1b9	1.115662313
Klk1b27	1.482036453
Cyp26b1	1.54764975
Zbtb16	1.977254743
Olfr461	1.885880503
Fv1	1.392069364
Tmppe	1.10428149
Hist2h3c2	1.803258596
Nat8f7	1.174465109
Zfp882	1.169763499
Fap	-1.825885902
Grasp	-1.298875768
Sult5a1	-1.809632128
Fkbp10	-1.036489408
Tcirg1	-1.025715402
Vsig2	-1.109324524
Srpk3	-1.561487227
Ltbp2	-2.667399518
Rec8	-1.241939578
Akap8l	-1.464340942
Hdgfl2	-1.036543743
Arrdc2	-1.234269394
Sult2b1	-2.61375313
Bax	-1.107093001
Col5a3	-1.743050522
Cacna1e	-1.93035737
Atn1	-1.577035152
Etfb	-1.863060023
Dnah2	-1.527437363
Fam131c	-1.433395577
Epor	-1.191075114
Slc4a3	-1.328847763
Zfp184	-1.093938962
Atp1a2	-1.040071899
Tssk4	-1.594024438
Cpt1c	-1.369340732
Pou2f2	-1.14535695
Dqx1	-1.094495064
Nav1	-1.06778796
Brf1	-1.015612206
Fuz	-1.299587899

Enkd1	-1.500205407
Clip3	-1.061891977
Hoxa2	-1.638000413
Slc9a5	-1.223360747
Aldh1a3	-1.772804774
Cyp2j9	-1.86737357
Gzmb	-1.21147218
Ager	-1.672204562
Anxa9	-1.467701366
Dhx58	-1.018112028
Mybl2	-1.831750737
Pcgf2	-1.210074706
Ybx2	-1.449166894
Dnah11	-1.325636957
Chtf18	-1.541739767
Ptger1	-2.251470106
Arhgef25	-1.208255345
Socs2	-1.464152545
Pcsk4	-1.488915525
Rab36	-1.107519088
Coro6	-2.048957681
Lrrc46	-1.417362763
Per1	-1.480139717
Pole2	-1.013588275
Tfap2a	-1.064873695
Gadd45g	-1.505268192
Fam193b	-1.477291476
Tert	-1.116385009
Mss51	-1.641351695
Bmp1	-1.204324265
Dok2	-1.21875599
Dscc1	-1.940740908
Apod	-1.487777661
Adck5	-1.057027118
Lypd2	-1.503175991
Mapk12	-1.332162529
Chkb	-1.403822028
Hira	-1.082188772
Prpf40b	-1.340435704
Aqp2	-1.005706803
Npff	-2.53982141
Cldn6	-1.648653649
Pkmyt1	-1.615402683

Satb1	-1.000808315
E4f1	-1.031744065
Rgs11	-1.858928769
Map3k8	-1.38078075
Adamts10	-1.213817007
Gabbr1	-1.593837844
Slc4a9	-1.256887129
Rps6kb2	-1.232184787
Ctsw	-1.499938059
Hells	-1.397015016
Lrrc45	-1.252188696
Pfkfb1	-1.06865089
Gli1	-1.933930405
Mbd6	-1.056830538
Agap2	-1.41275089
Adam8	-1.381046868
Irf7	-1.052906831
Lmntd2	-1.44275621
Pidd1	-1.170037329
Col7a1	-1.581424825
Asnsd1	-1.380775675
Pkn3	-1.273055347
Dnm1	-1.31632035
Nr4a2	-1.434443418
Card9	-1.192405132
Gpsm1	-1.323490525
Syt13	-1.138593196
Hdc	-2.155541361
Mall	-1.457465107
Ccm2l	-1.398435279
S100a11	-1.099967016
Efna4	-1.543823637
Celf3	-2.372879313
Gem	-1.205937407
Ambp	-1.767452962
Npr2	-1.022937503
Mfsd2a	-1.672859539
Mutyh	-1.204084289
Ccdc163	-1.087923874
Miip	-1.158735908
Tnfrsf4	-2.580082234
Cenpa	-1.26269982
Nsun7	-1.692644086

Uchl1	-1.413944447
Slc10a6	-2.369554311
Cabp1	-1.405725769
Rasal1	-2.642186344
Tmem213	-1.331942125
Nfe2l3	-1.120637852
Grip2	-1.260291957
Lrmp	-1.020737005
Ing4	-1.710746136
Tgfb1i1	-1.242509679
Itgam	-1.966280955
Cd37	-1.236949675
Dnhd1	-1.087966191
Praf2	-1.305456353
Gabre	-1.539403435
Plp1	-1.04314245
Pllp	-1.440993993
Chek1	-1.211791439
Robo3	-1.177680455
Pde4a	-1.006776912
Dock6	-1.230501215
Kif23	-1.143851182
Csrnp1	-1.134453434
Cish	-1.407762231
Klhdc8b	-1.354271325
Oas2	-1.538864679
Mdfi	-1.30676517
Abcc10	-1.006748654
Rnft2	-1.040705841
Inha	-1.855291478
Znrf1	-1.175562033
Tspoap1	-1.187734141
Ly6d	-1.827243211
Cacna1a	-1.223644496
Tle2	-1.532095729
Cpne7	-1.335760556
Ncaph	-1.670003372
Ushbp1	-1.055556884
Igsf6	-1.043919556
Ssc5d	-2.006086155
Caps2	-1.593222794
Arhgap45	-1.339968394
Usp35	-1.439631331

H2-Q4	-1.388408468
Zfp57	-1.638419029
Phf24	-1.650615113
Slc2a6	-1.413141919
Fxyd1	-1.24435299
Zfp692	-1.598633625
Aldh3b3	-1.327978029
Mfsd2b	-1.26483761
Cep85	-1.366176482
Plekhg2	-1.308671876
Wdhd1	-1.09424353
Kcnk2	-1.348718928
Hoxb5	-1.070460478
A230050P20Rik	-1.74487139
Irs2	-1.018578053
Zfp653	-1.502593059
Nexn	-1.225517596
Isg20	-1.208092551
Dusp10	-1.290042677
Neil3	-1.75857586
Ncam1	-1.177219596
Timeless	-1.034433124
Plcb2	-1.026269445
Arntl2	-1.104390142
Tbc1d10c	-1.543169903
Cchcr1	-1.10650829
Dtx3	-1.151257675
Hipk4	-1.774443512
Xaf1	-1.279078861
Plppr2	-1.813505784
Cd79b	-1.458023364
Col16a1	-1.019822786
Myh6	-2.19319365
Arhgef1	-1.113721015
Tspyl2	-1.055435252
Rgl2	-1.018320889
Meis3	-1.655591669
Serpina3g	-1.698321434
H2-Ob	-1.136045074
Lrrc29	-1.059617837
Mapk1ip1	-1.359797537
Mcm3	-1.116369198
Nfkbil1	-1.317570066

Ypel3	-1.073842881
Olfr78	-1.827795206
Fam25c	-1.423819555
Als2cl	-1.013908757
Il20rb	-2.303732639
Tceal3	-1.08570211
Tex38	-2.98328
Lrrc3b	-1.417075049
Penk	-1.381583264
Scand1	-1.495680672
Ankle1	-1.823440635
Mroh7	-1.268379218
Palm3	-1.014643867
Tmem81	-1.200188171
Cracr2b	-1.31679872
Ccdc57	-1.217351164
Nrap	-1.105420989
Tmem200a	-2.899345421
Grem2	-3.030951008
Grrp1	-1.016161743
Tmc8	-1.478415536
Eva1b	-1.277994014
Carmil2	-1.68618158
Zfp579	-1.740152799
Kcnf1	-1.060749886
Kctd14	-1.541379279
Nlgn2	-1.422682026
Serp2	-1.628322793
A630001G21Rik	-1.499149718
Mapk11	-1.334343003
Fes	-1.079963029
Ebf4	-1.710042381
Lgals4	-1.428957118
Ankrd24	-1.147332937
Tmem158	-1.605920744
Zfp580	-1.098549306
Izumo4	-1.264285718
Fam19a3	-1.878669977
Tmem150a	-1.062724529
Csf2ra	-1.156992717
Tcea2	-1.045321899
Nrm	-1.35004934
Arrb2	-1.019558837

Mfap2	-1.358740033
Ifitm2	-1.107073141
Eno3	-1.286836565
Clasrp	-1.151305034
Ccdc62	-1.617904382
Serpina10	-1.764883771
Lmtk3	-3.412176165
Tubb4a	-1.31548817
Rpl35	-1.09560297
Egfem1	-2.661695006
Slc22a21	-1.107531455
Mapk15	-1.575152457
mt-Nd6	-1.080778022
Fbxl12	-1.042659137
Mas1	-1.178487721
Il3ra	-1.803693042
Tmem100	-1.642151635
Naip5	-1.19683818
Csf2rb2	-1.149448931
Eme2	-1.088567673
Ecscr	-1.072675385
Rpl27-ps3	-1.611868748
Sh3d21	-1.11409455
Bglap3	-1.497842789
Ripor3	-1.182650723
Sptbn5	-1.435645445
Hoxc4	-1.447106729
Nrbp2	-1.674675673
Rad54b	-1.818096208
Prkcg	-1.987741341
Insl3	-1.265547945
Kcnip3	-1.430631711
Hoxd3	-1.520870979
Phyhd1	-1.307631809
Tmsb10	-1.154871
Stard6	-1.889127516
Rnf8	-1.341726217
Itga10	-1.55876101
Churc1	-1.718123567
Prr22	-2.610973129
Col6a5	-1.408580385
Gm20518	-1.726100063
Higd1c	-1.447022529

Tnfsf12	-1.326280858
Xndc1	-1.090155466
Gtf3c2	-1.025632578
Flt3l	-1.57867425
Lilr4b	-1.178866943
